# A clinically relevant model of acute respiratory distress syndrome in human-size swine

**DOI:** 10.1242/dmm.049603

**Published:** 2022-10-10

**Authors:** Sarah R. Kaslow, Jonathan A. Reimer, Meghan R. Pinezich, Maria R. Hudock, Panpan Chen, Mariya G. Morris, Mandy L. Kain, Jay S. Leb, Carrie B. Ruzal-Shapiro, Charles C. Marboe, Matthew Bacchetta, N. Valerio Dorrello, Gordana Vunjak-Novakovic

**Affiliations:** ^1^ Department of Surgery, Columbia University Medical Center, New York, NY 10032, USA; ^2^ Department of Biomedical Engineering, Columbia University, New York, NY 10032, USA; ^3^ Department of Surgery, Mount Sinai Hospital, Chicago, IL 60608, USA; ^4^ Vagelos College of Physicians and Surgeons, Columbia University Medical Center, New York, NY 10032, USA; ^5^ Institute of Comparative Medicine, Columbia University Medical Center, New York, NY 10032, USA; ^6^ Department of Radiology, Columbia University Medical Center, New York, NY 10032, USA; ^7^ Department of Pathology, Columbia University Medical Center, New York, NY 10032, USA; ^8^ Department of Thoracic Surgery, Vanderbilt University Medical Center, Nashville, TN 37232, USA; ^9^ Department of Pediatrics, Columbia University Medical Center, New York, NY 10032, USA; ^10^ Department of Medicine, Columbia University Medical Center, New York, NY 10032, USA

**Keywords:** Animal model, Lung, Inflammation, Systemic, Pathophysiology, Pulmonary function

## Abstract

Despite over 30 years of intensive research for targeted therapies, treatment of acute respiratory distress syndrome (ARDS) remains supportive in nature. With mortality upwards of 30%, a high-fidelity pre-clinical model of ARDS, on which to test novel therapeutics, is urgently needed. We used the Yorkshire breed of swine to induce a reproducible model of ARDS in human-sized swine to allow the study of new therapeutics, from both mechanistic and clinical standpoints. For this, animals were anesthetized, intubated and mechanically ventilated, and pH-standardized gastric contents were delivered bronchoscopically, followed by intravenous infusion of *Escherichia coli*-derived lipopolysaccharide. Once the ratio of arterial oxygen partial pressure (PaO_2_) to fractional inspired oxygen (F_I_O_2_) had decreased to <150, the animals received standard ARDS treatment for up to 48 h. All swine developed moderate to severe ARDS. Chest radiographs taken at regular intervals showed significantly worse lung edema after induction of ARDS. Quantitative scoring of lung injury demonstrated time-dependent increases in interstitial and alveolar edema, neutrophil infiltration, and mild to moderate alveolar membrane thickening. This pre-clinical model of ARDS in human-sized swine recapitulates the clinical, radiographic and histopathologic manifestations of ARDS, providing a tool to study therapies for this highly morbid lung disease.

## Teaser

**Summary:** A pre-clinical model of acute respiratory distress syndrome (ARDS) in human-sized swine, which recapitulates the clinical, radiographic and histopathologic manifestations of ARDS, providing a tool to study therapies for this highly morbid lung disease.

## INTRODUCTION

Acute respiratory distress syndrome (ARDS) is a highly lethal pattern of lung injury, with hospital mortality rates of 35-46%, depending on severity (Bellani et al., 2016). Despite more than 30 years of clinical trials, targeted therapeutics with proven mortality benefits remain elusive (Matthay et al., 2017; Shaw et al., 2019). Evidence to support adjunctive treatments, including extracorporeal membrane oxygenation (Combes et al., 2018; Peek et al., 2009), have remained equivocal. Thus, the treatment of ARDS is supportive in nature, relying on therapies aimed at improving ventilation and oxygenation, and limiting further lung damage, such as lung protective ventilation (Acute Respiratory Distress Syndrome Network et al., 2000), prone positioning and neuromuscular blockade (Papazian et al., 2010) while waiting for the patient to recover. Examining novel, targeted therapeutics, from both mechanistic and clinical standpoints, using a pre-clinical model with direct clinical relevance, therefore, remains a research priority for the National Heart, Lung, and Blood Institute (NHLBI) and American Thoracic Society (ATS) (Semler et al., 2020; Shaw et al., 2019).

The precise clinical criteria of ARDS diagnosis provided by the Berlin Definition (ARDS Definition Task Force et al., 2012) (Table S1), routinely used clinically and applied to human-sized models, allows for critical evaluation of animal models for clinical relevance. Key features of an experimental model of acute lung injury in animals include (1) rapid onset after the inciting event, (2) strong evidence of physiologic dysfunction, (3) robust inflammatory response, (4) alteration in the alveolar-capillary barrier and, (5) histologic evidence of tissue injury (Matute-Bello et al., 2011; Semler et al., 2020). Large animal models of ARDS induced via repeated broncheoalveolar lavage (Araos et al., 2016), oleic acid (Borges et al., 2019) or endotoxin infusion (Nieman et al., 1996), gastric acid aspiration (Meers et al., 2011a) and inhalation injury (Leiphrakpam et al., 2021) have been reported, with each model mimicking certain aspects of the human disease to varying degrees (Ballard-Croft et al., 2012). However, few of these models rely on multifactorial etiologies of acute lung injury to both sides of the alveolar-capillary interface (Tiba et al., 2021) and few have been comprehensively evaluated regarding clinical, radiographic and histopathologic features.

We hypothesized that dual-hit injury to the epithelium and endothelium of the lung in human-sized swine can recapitulate moderate to severe human ARDS clinically, radiographically and histopathologically. In line with NHLBI and ATS research priorities, the goal of this study was to identify a reproducible method for inducing ARDS over an appropriate time scale to allow studies of new therapeutics within the context of organ dysfunction, organ support and co-interventions available to human patients. Such a pre-clinical model is crucial to maximize the utilization of resources in clinical trials.

## RESULTS

### Oxygenation impairment and clinical progression

**Fig. 1.**
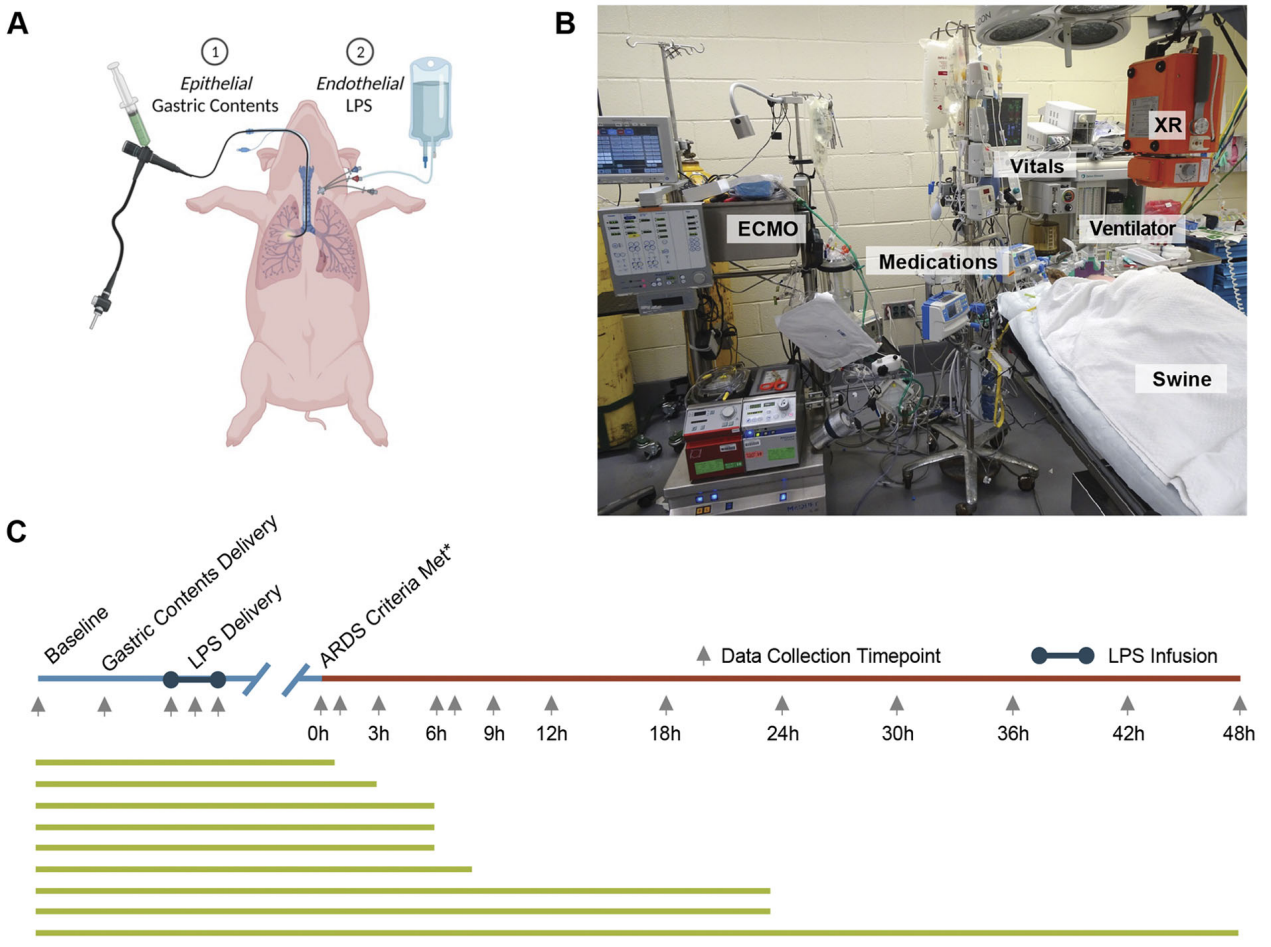
**Experimental overview.** (A) Schematic of ARDS induction: epithelial lung injury induced by bronchoscopic delivery of 30-50 ml of standardized (pH 2.0) gastric contents to bilateral lungs, followed by central venous infusion of LPS. (B) Set-up of the operating room. (C) Data collection and experimental time course; green lines represent animal experiments. LPS, lipopolysaccharide; ABG, arterial blood gas; ECMO, extracorporeal membrane oxygenator; XR, X-ray machine.

We induced ARDS in the Yorkshire breed of swine by injuring the lungs of these animals with gastric acid and through infusion of endotoxin. For this, swine (*n*=9) were anesthetized, intubated and mechanically ventilated. Dual-hit injury induction was achieved by bronchoscopic delivery of standardized gastric contents (pH 2) followed by intravenous infusion of *Escherichia coli*-derived lipopolysaccharide. When the ratio of arterial oxygen partial pressure (PaO_2_ in mm Hg) to fractional inspired oxygen (F_I_O_2_) had decreased to <150 – hereafter referred to ARDS 0 h – swine received standard ARDS treatment for up to 48 h. All swine developed moderate to severe ARDS (minimum PaO_2_:F_I_O_2_ ratio of 80±17). This produced a moderate to severe oxygenation impairment, i.e. a decrease in the ratio of arterial oxygen partial pressure (PaO_2_ in mm Hg) to fractional inspired oxygen (F_I_O_2_) (minimum PaO_2_:F_I_O_2_ ratio: 63-112), that failed to resolve (i.e. PaO_2_:F_I_O_2_>300 on minimal ventilator settings) until at least 48 h after induction (see Fig. 1A, for experimental overview). Baseline measurements and characteristics of all animals were similar (Table S2). Following dual-hit injury induction, all nine animals met the diagnostic criteria for ARDS with an average minimal PaO_2_:F_I_O_2_ ratio of 80±17. On arterial blood gas, the mean PaO_2_ was significantly lower at ARDS 0 h (90.3±27.2) relative to that at baseline (497.5±38.3, *P*<0.001). Of the nine animals, three died prior to the planned study endpoint, i.e. at 2 h, 3 h or 8 h after onset of ARDS (ARDS 2 h, ARDS 3 h or ARDS 8 h, respectively) caused, respectively, by a massive pulmonary embolus, severe hypoxemia or a dislodged endotracheal tube, all resulting in hypoxic cardiopulmonary arrest. Although we observed variations between animals, the average time until ARDS 0 h – i.e. PaO_2_:F_I_O_2_ <150 following the completion of LPS infusion – was 1.5±1.5 h (Table S3). The PaO_2_:F_I_O_2_ ratio remained <300, with minimal ventilator settings for ARDS 6 h endpoint animals (*n*=3) and ≤48 h. Owing to severely impaired oxygenation without improvement upon lung protective ventilation and/or paralytics, five animals required extracorporeal membrane oxygenation (ECMO).

**Fig. 2.**
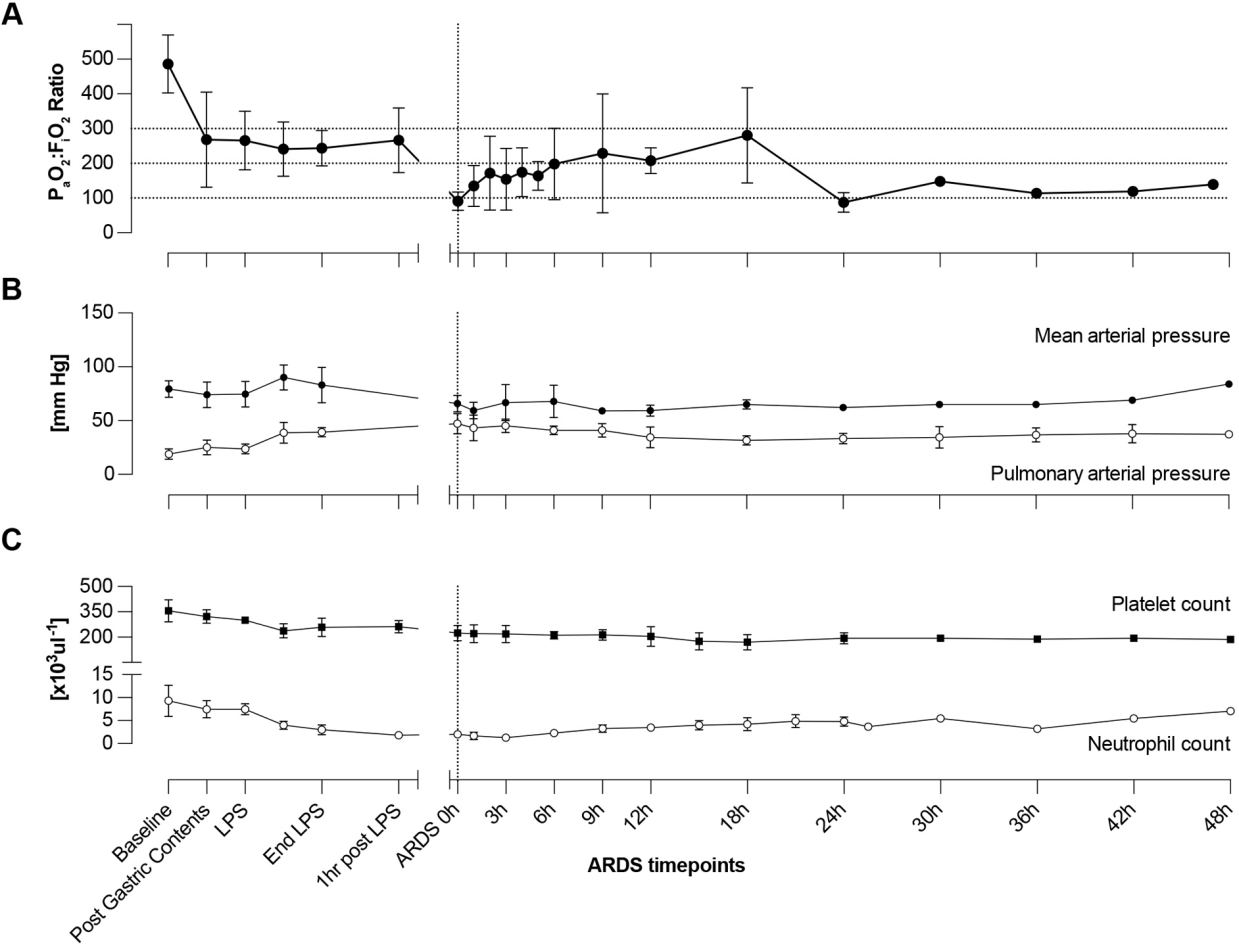
**Clinical measurements over experimental time course.** (A) P_a_O_2_:F_i_O_2_ ratio as measured by arterial blood gas over experimental time course. (B) Mean arterial pressure (black) and pulmonary arterial pressures (open circles) in millimeters of mercury (mm Hg) over experimental time course. (C) Platelet (black circles) or neutrophil counts (open circles) [×10^3^μl^−1^] over the experimental time course (in hours).

ARDS induction was associated with arterial hypotension, pulmonary hypertension and respiratory acidosis. All animals developed arterial hypotension and seven out of nine animals required vasopressors to maintain a mean arterial blood pressure of >55 mm Hg. At ARDS 0 h, mean arterial pressure was below that of baseline (66±8 mm Hg versus 80±8 mm Hg) (Fig. 2), whereas pulmonary arterial pressure at ARDS 0 h was significantly elevated compared to baseline (47±10 mm Hg versus 19±5 mm Hg; *P*<0.001), primarily driven by hypoxemic and hypercapnic pulmonary vascular resistance, and minimally responsive to ventilator manipulation. Mean arterial pH at ARDS 0 h was significantly lower than mean arterial pH at baseline (7.318±0.086 versus 7.455±0.052; *P*=0.002) with an associated increase in PaCO_2_ at ARDS 0 h (52±7 versus 46±5; *P*=0.156). Pulmonary arterial pressure remained elevated until the experimental endpoint (Fig. 2).

ARDS induction was also associated with a significant decline in the number of circulating neutrophils compared to those at baseline (2.0±0.7×10^3^μl^−1^ versus 9.3±3.4×10^3^μl^−1^; *P*=0.001). In six out of eight animals, neutrophil counts reached a minimum between ARDS 0 h and ARDS 3 h (Fig. S2B, Fig. S3B, Fig. S4B, Fig. S5B, Fig. S6B, Fig. S7B, Fig. S8B, Fig. S9B). On average, numbers of circulating platelets also decreased following ARDS induction compared to those at baseline (179±70×10^3^μl^−1^ versus 356±66×10^3^μl^−1^).

**Fig. 3.**
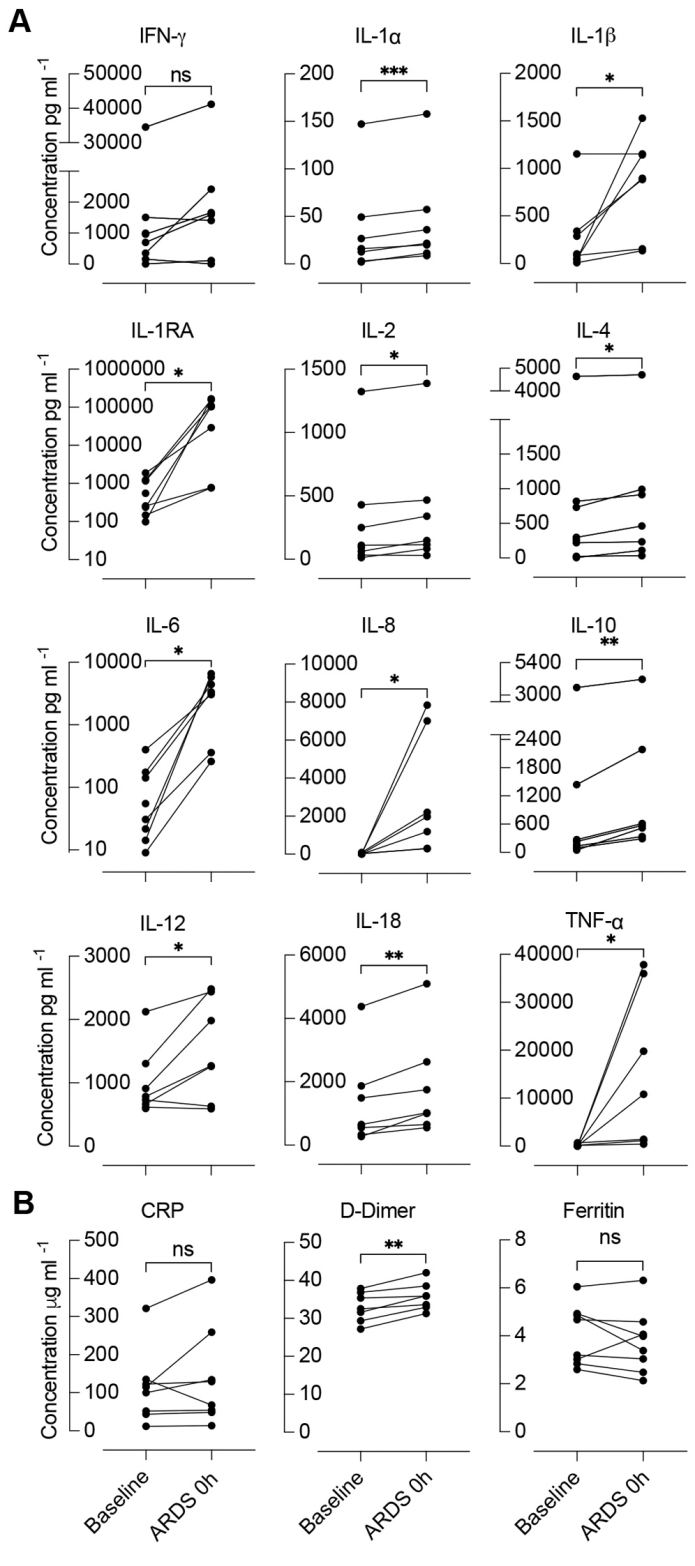
**Change in cytokine and acute-phase reactants from baseline to induction of ARDS.** (A) Cytokine levels at baseline and ARDS 0 h (*n*=7). (B) Acute-phase reactants at baseline and ARDS 0 h (*n*=7). ns, not significant; *P<0.05; **P<0.01; ***P<0.001. CRP, C-reactive protein. *x*-axis labeling shown in B also applies to all graphs in A.

Plasma cytokines were measured in duplicate at baseline and ARDS 0 h. At ARDS 0 h, levels of pro-inflammatory cytokines, i.e. interleukins IL1A, IL2, IL6, IL12 and IL18 (hereafter referred to as IL-1α, IL-2, IL-6, IL-12, IL-18, respectively) and tumor necrosis factor alpha (TNF-α) were significantly elevated relative to baseline (Fig. 3A). Additionally, concentrations of IL1B (hereafter referred to as IL-1β) and its receptor antagonist IL1RN (also known as IL-1RA) were significantly increased at ARDS 0 h (134-1527 pg ml^−1^, *P*=0.038 and 770-166,323 pg ml^−1^, *P*=0.024). Plasma concentration of the pro-inflammatory chemoattractant IL-8 was significantly elevated (249-7839 pg ml^−1^). Levels of anti-inflammatory cytokines (IL-4 and IL-10) were also significantly increased at ARDS 0 h (36-4711 pg ml^−1^, *P*=0.0234 and 294-4164 pg ml^−1^; *P*=0.0017). Only levels of interferon gamma (INFG; hereafter referred to as IFN-γ) were not significantly increased.

Acute-phase reactants (C-reactive protein, D-dimer, ferritin) were measured in blood plasma or serum by using ELISA (Table S5). Levels of D-dimer were significantly increased at ARDS 0 h in all animals (*P*=0.004) (Fig. 3B). Although not significant when compared as an average, C-reactive protein was elevated from baseline in all but one animal (Fig. 3B).

### Radiographic evidence of lung edema

**Fig. 4.**
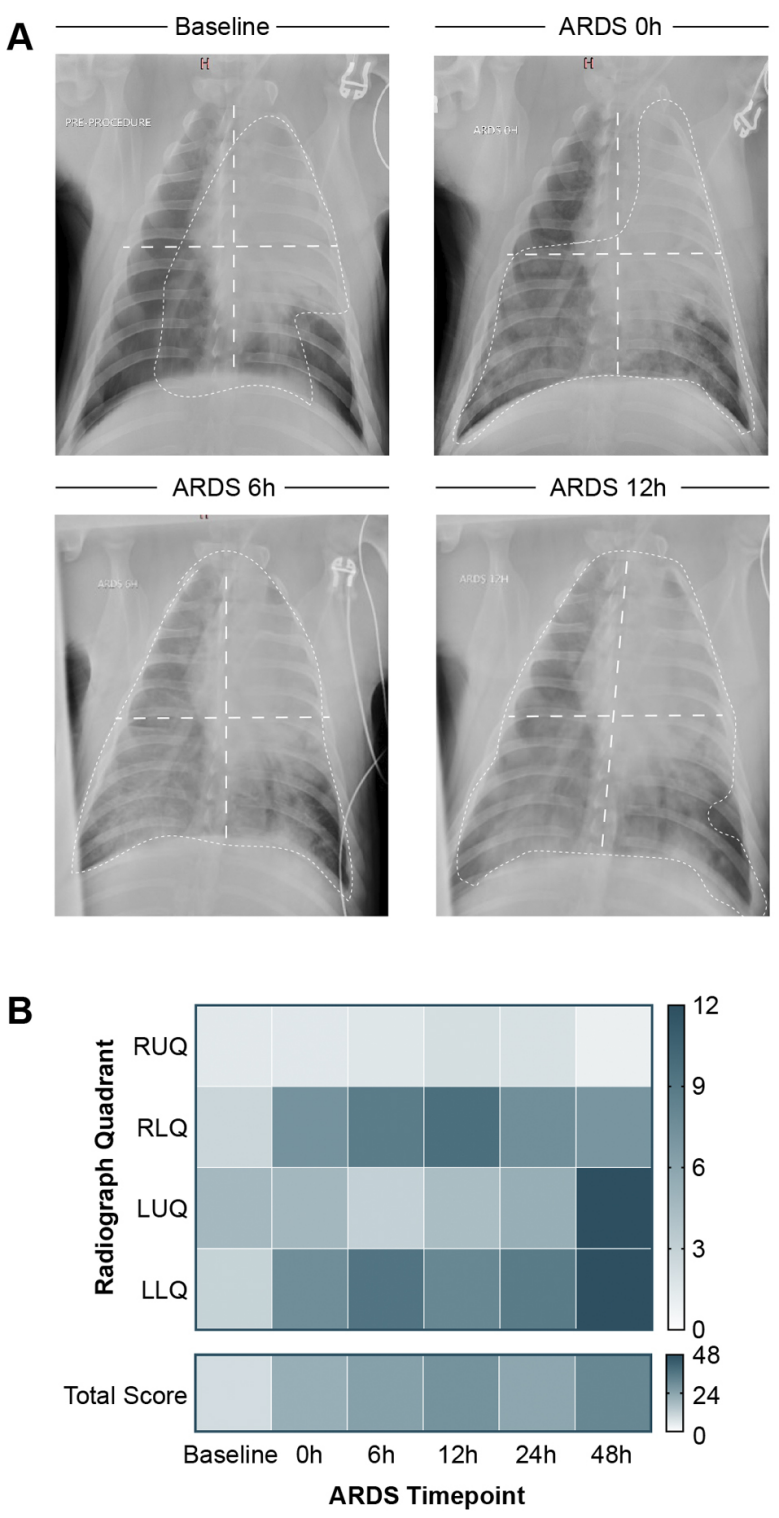
**Radiographic progression of ARDS.** (A) Representative chest X-rays from baseline up to ARDS 12 h demonstrate radiographically the progression of ARDS (long dashed white lines, approximate delineation of lung field quadrants; short dashed white lines, area of radiographic edema). (B) Median radiographic assessment of lung edema score per radiographic lung field quadrant and as total for ‘baseline’ lung (*n*=9), ARDS 0 h (*n*=7), ARDS 6 h (*n*=5), ARDS 12 h (*n*=3), ARDS 24 h (*n*=3), ARDS 48 h (*n*=1). RUQ, right upper quadrant; RLQ, right lower quadrant; LUQ, left upper quadrant, LLQ, left lower quadrant. Color scale from white (0) to dark blue (12, individual lung field quadrant; 48, total score).

Interval chest X-rays demonstrated progressive bilateral opacification of the lung fields, consistent with the radiographic component of the Berlin criteria for ARDS diagnosis (Table S1, Fig. 4) (ARDS Definition Task Force et al., 2012). Semi-quantitative RALE scoring (as described in Warren et al., 2018) by two independent reviewers, interobserver agreement by Pearson's correlation coefficient (r=0.88, 95% CI 0.82-0.92, *P*<0.001; see Fig. S10A) and Bland-Altman plot (Fig. S10B) were high. Mean lung edema scores increased compared to baseline (11±7) at ARDS 0 h (21±7, *P*=0.005; Fig. 4). Lung edema persisted after ARDS induction through endpoint and negatively correlated with the PaO_2_:F_I_O_2_ ratio (*P*<0.001). Lung edema was significantly worse in lower lung quadrants versus upper lung quadrants at ARDS 0 h (RALE score: 7.7±2.1 versus 2.9±2.8, *P*=0.016) and 6 h (9.1±2.0 versus 2.3±2.0, *P*<0.001) but not significantly different at ARDS 12 h (8.8±3.0 versus 3.3±2.5, *P*=0.114) and ARDS 24 h (8.3±0.4 versus 3.5±2.5, *P*=0.254) (Fig. 4; Fig. S11).

### Histopathologic analysis

**Fig. 5.**
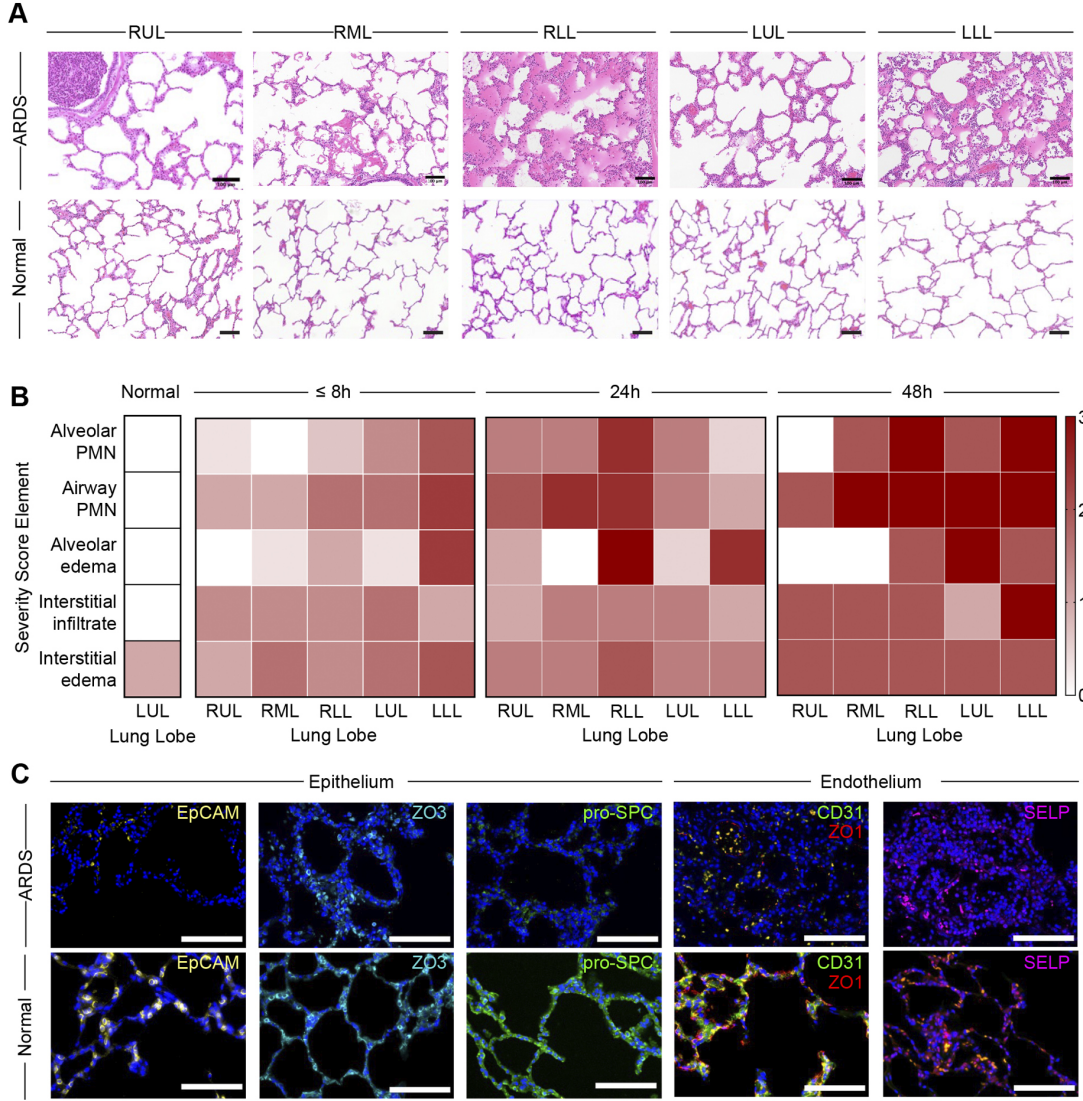
**Histopathologic analysis.** (A) Representative H&E slides from ARDS and normal swine lungs arranged by lung lobe. (B) Lung injury severity score by severity score element and lung lobe by study endpoint (≤8 h, *n*=3; 24 h, *n*=2; 48 h, *n*=1). Color scale from white (normal) to red (severe injury). (C) Immunofluorescence images of ARDS and normal swine lungs stained for the epithelial markers EpCAM, ZO3 and pro-SPC, and the endothelial markers (CD31, ZO1 and SELP). RUL, right upper lobe; RML, right middle lobe; RLL, right lower lobe; LUL, left upper lobe; LLL, left lower lobe; PMN, polymorphonuclear cells. Scale bars: 100 μm (A), 50 μm (C).

H&E slides of experimental and normal swine lung tissue were randomly numbered prior to pathologic review under light microscope by an experienced pulmonary pathologist. A previously described lung-injury severity score, which includes the number of airway polymorphonuclear (PMN) cells per high-power field (hpf), the number of alveolar polymorphonuclear cells per hpf, alveolar edema, interstitial infiltrates (lymphocytes and neutrophils in the interstitium around vessels and airways and in alveolar septa and pleura) and interstitial edema (perivascular and peribronchial spaced expanded with edematous fluid), as described in O’Neill et al. (2017), was applied (Fig. S1D, Fig. S2E, Fig. S6E, Fig. S7E, Fig. S8E, Fig. S9E, Table S7). Minimal and maximum scores for each sub-score element are 0 and 3, respectively (except interstitial edema, for which the maximum sub-score is 2). The average total lung injury severity score was 1.0±0.0 for normal swine lungs, whereas the mean total lung injury severity score was 6.1±1.8 at ARDS <8 h, 7.7±0.4 at ARDS 24 h, and 10.2±2.8 at ARDS 48 h. Among score components, the interstitial edema and airway PMN sub-scores were highest for all experimental timepoints. Animals demonstrated time-dependent increases in interstitial edema (ARDS <8 h: average 1.5±0.1; ARDS 24 h: average 1.6±0.3; ARDS 48 h: average 2.0±0.0) and alveolar edema sub-scores (ARDS <8 h: average 0.8±0.7; ARDS 24 h: average 1.4±0.0; 48 h: average 1.4±1.3, Fig. 5).

There was no significant difference in total lung injury severity score between left and right lower lobes (LLL and RLL, respectively) and non-lower lobes, i.e. right upper lobe (RUL), right middle lobe (RML) and left upper lobe (LUL) at ARDS <8 h (*P*=0.0838), ARDS 24 h (*P*=0.1361), or ARDS 48 h (*P*=0.1387). However, lower lobes demonstrated higher alveolar edema sub-scores at ARDS <8 h (*P*=0.0103) and ARDS 24 h (*P*=0.0014) but not at ARDS 48 h (*P*=0.2191). Hyaline membranes were not noted at study endpoint.

Immunofluorescence imaging showed decreased markers of both epithelial cell and endothelial cell adhesion [EpCAM and P-selectin (SELP), respectively] in lungs from animals with ARDS relative to those from healthy controls (Fig. 5C). Immunostaining for surfactant protein C (SFTPC; also known as pro-surfactant protein C, pro-SPC) a marker of type 2 alveolar epithelial cells (AT2), showed reduced immunofluorescence in lung tissue from ARDS swine compared to that in normal swine tissue. Markers of tight junctions and epithelial-endothelial barrier integrity (i.e. ZO3 and ZO1) were similarly decreased.

## DISCUSSION

In this present study, we aimed to recapitulate the clinical, radiographic and histopathologic features of acute respiratory distress syndrome (ARDS) in a pre-clinical model to facilitate the study of potential therapeutics for this increasingly frequent and severe lung disease. We modeled the pathophysiology of ARDS by using two clinically relevant injuries (gastric acid aspiration and endotoxin infusion) and fulfilled the criteria delineated by the NHLBI and ATS for representative ARDS model (Matute-Bello et al., 2011). We addressed the critical gap in the need for pre-clinical models that can be used to deepen our understanding of the pathophysiology of ARDS, identify biomarkers of each phase of disease and test emerging targeted therapies.

ARDS induction by gastric acid injury and endotoxin reliably produced moderate to severe oxygenation impairment that failed to resolve up to 48 h after induction, suggesting that the lung injury is not merely a transient pneumonitis. Rapid onset after the inciting event is a key pre-model feature. One recently published swine model utilizing both direct and indirect lung injury reached a PaO_2_:F_I_O_2_ ratio of ≤300 by ARDS 12 h; however, the mean PaO_2_:F_I_O_2_ ratio was above the threshold for severe ARDS (Tiba et al., 2021). The induction timeframe in that model (i.e. with PaO_2_:F_I_O_2_ ratios consistent with moderate to severe ARDS within 1.5±1.5 h following infusion of LPS) mimics the clinical progression of ARDS in humans and aligns with the temporal diagnostic criterion of ARDS (ARDS Definition Task Force et al., 2012). In one large single-institution study, human patients with early-onset ARDS developed ARDS within 4.2 h, the interquartile range being 2.3-7.4 (Fuchs et al., 2019).

We also observed strong evidence of physiologic dysfunction beyond oxygen impairment. All hemodynamic responses and changes in laboratory values seen after intravenous infusion of LPS (i.e. leukopenia, decreased cardiac output and arterial hypotension) (Matute-Bello et al., 2008) were observed, as were significant increases in pulmonary arterial pressure. Because the model of ARDS presented here involves human-sized animals, measurement and monitoring of these parameters within an intensive-care-unit level of care is possible, circumventing the practical difficulties of repeated blood sampling and prolonged organ support that limit the applicability of small animal models. To confirm the alterations in the inflammatory response are consistent with ARDS, glucocorticoids were not administered (Meduri et al., 1995). Furthermore, interventions, such as prone positioning, vasopressors, crystalloid fluid administration, neuromuscular blockade and ECMO, were co-administered in a manner consistent with that provided to humans. An advantage of this model is the ability to test candidate treatments in a clinical context, thereby mimicking that of human patients.

Neutrophils are central mediators of the pathogenesis of acute lung injury and crucial for the robust inflammatory response of ARDS (Abraham et al., 2000; Folkesson et al., 1995). Excessive neutrophil recruitment to the lungs and neutrophil activation contribute to the progression of ARDS through damage of bystander tissue and further loss of lung function (Grommes and Soehnlein, 2011; Williams and Chambers, 2014). Our finding that levels of circulating neutrophils significantly decrease (69-93%) between ARDS 0 h and ARDS 6 h (Fig. 2C) suggests that neutrophils are largely sequestered within the pulmonary vasculature, parenchyma and airways. We found that accumulation of neutrophils within the alveoli and airways in lung tissues worsened over the experimental time course (Fig. 5), i.e. at all three histopathologic analysis timepoints after ARDS 0 h lung tissue contained on average >300 PMN cells per hpf compared to 22 PMNs per hpf in control lung specimens. Additionally, levels of IL-8 – a potent neutrophil chemoattractant (Grommes and Soehnlein, 2011; Williams and Chambers, 2014) and suggested biomarker for ARDS (Whitney et al., 2020; Yadav et al., 2018) – were significantly elevated from baseline at ARDS 0 h in our experimental model (Fig. 3).

Other elements of the inflammatory response also developed after dual-hit lung injury. As previously demonstrated in patients with ARDS, levels of pro-inflammatory cytokines in the experimental animals were significantly elevated at ARDS 0 h relative to those at baseline (Fig. 3) (Meduri et al., 1995; Puneet et al., 2005; Schütte et al., 1996). Plasma concentrations of IL-1β and its receptor antagonist IL-1RA, both of which were significantly elevated after ARDS induction in our model, correlated with ARDS severity and have been associated with clinical outcomes (Meduri et al., 1995).

Alteration of the alveolar-capillary barrier and development of alveolar edema are key pathophysiologic features of ARDS. Two independent reviewers semi-quantitatively analyzed lung edema by chest radiography, and found that mean lung edema had increased from baseline to ARDS 0 h, correlating with worsening oxygenation (Fig. 4). This alteration of the alveolar-capillary barrier was further demonstrated by histopathologic analysis. Diffuse alveolar injury – i.e. the near pathologic correlate to the clinical entity of ARDS – involves the accumulation of neutrophils in alveolar or interstitial spaces, formation of hyaline membranes, thickening of the alveolar wall and enhanced injury (Cardinal-Fernandez et al., 2017; Matute-Bello et al., 2011). In our study, we did not observe hyaline membranes in most swine. But Cardinal-Fernandez et al. also found hyaline membranes and diffuse alveolar damage (DAD) in only half of human patients undergoing open-lung biopsies for clinical ARDS (Cardinal-Fernandez et al., 2017). The predominant lack of hyaline membranes in the swine model might relate to the timescale of our study or the peripheral location of the lung specimens. Other pathologic features of the acute exudative phase of ARDS (i.e. of diffuse alveolar damage, interstitial and alveolar edema, inflammation, and fibrin deposition) were observed with increasing severity of lung injury over the experimental time course (Fig. 5).

Other swine models have examined alternative injury-induction methods, i.e. repeated saline lavage together with ventilation injury (Araos et al., 2021, 2016), volutrama and hyperoxia followed by direct inoculation of *Escherichia coli* by acidified gastric particles (Tiba et al., 2021). Most ARDS animal models have demonstrated injury to the lung epithelium or changes in the pulmonary vascular compartment, including hypertension and the formation of microthrombi (Araos et al., 2016; Borges et al., 2019; Nieman et al., 1996; Meers et al., 2011a; Leiphrakpam et al., 2021), but few have demonstrated alterations to both sides of the alveolar-capillary interface in a manner consistent with ARDS in human patients (Millar et al., 2020). The sheep model used by Millar and colleagues enables the mechanistic study of ARDS pathophysiology and is highly suitable for developing and evaluating therapeutics because it recapitulates the clinicopathologic features of injury to both the epithelium and endothelium in ARDS.

*Ex vivo* models, in which human lungs were injured by intrabronchial instillation of endotoxin (Lee et al., 2009), have also been used for pre-clinical testing of candidate therapies (Shaw et al., 2019). One pre-clinical model specifically evaluated permeability of the lung endothelial barrier and clearance of the alveolar fluid, and treatment with bone marrow-derived multipotent mesenchymal stem cells (MSCs) (Lee et al., 2009; Millar et al., 2020) and informed subsequent clinical trials in humans (Matthay et al., 2019). This phase 2a safety trial demonstrated the safety of allogeneic MSCs for patients with moderate to severe ARDS but did not evaluate its efficacy (Matthay et al., 2019). Because the swine model presented here comprehensively recapitulates ARDS within a clinically relevant context beyond the endothelial barrier, it is particularly well suited to evaluate dosing strategies and pre-clinical efficacy of candidate therapeutics, such as MSCs or their secretome, to maximize resources dedicated to clinical trials.

### Limitations

Variations in mechanism and intensity of lung injury may provide insights into the pathophysiologic mechanisms of ARDS (Beitler et al., 2022). Although acid aspiration is a reproducible mechanism of lung injury, the window between injurious and non-injurious doses is narrow (Matute-Bello et al., 2008), making tuning of the degree of injury and ARDS severity difficult. The animal-to-animal variability of this model accurately captures inter-patient variability but cannot detect small variations in biomarkers or clinical data. By their very nature, experimental models emulate simplified and tightly controlled clinical scenarios, a limitation that also applies to our study. We investigated the inflammatory response in the lungs to bronchoalveolar lavage. Our model was designed to mimic the clinical course and treatments experienced by human patients. However, due to the severe lung dysfunction that occurred by establishing the ARDS conditions in our model, we were unable to reliably perform bronchoscopy without compromising oxygenation and recruitment of the lungs. Furthermore, swine commonly have underlying lung disease and bacterial colonization, which may augment the severity of ARDS in this model.

Our model is resource intensive, requiring around-the-clock intensive care by trained medical and veterinary doctors. Additionally, the model allows for co-interventions (e.g. ECMO), which might not be available to all patients in all clinical settings, as it requires specialized equipment and staffing. Furthermore, a follow-up period of 48 h is a very short clinical timeframe in the context of the lengthy pathology of ARDS, particularly as it evolves over the course of weeks. Future studies focusing on the efficacy of novel therapeutics would require an extended experimental time course.

### Conclusion

This pre-clinical model in human-sized swine recapitulates the clinical, radiographic, and histopathologic manifestations of ARDS, providing a much-needed tool to study pathogenesis and targeted therapeutics for this highly morbid, and increasingly incident, lung disease.

## MATERIALS AND METHODS

Additional details regarding materials and methods are provided in the Supplementary Materials and Methods as indicated. All animals were treated in accordance with the Guide for the Care and Use of Laboratory Animals, 8th Edition and housed in facilities accredited by the Association for Assessment and Accreditation of Laboratory Animal Care International (AAALACI). All studies were approved by the Institutional Animal Care and Use Committee of Columbia University (National Research Council, 2011).

### Pre-procedure preparation

Female Yorkshire swine (49±5 kg, Table S2) were placed under intravenous general anesthesia after sedation with tiletamine/zolazepam. They were initially ventilated by using volume-control with tidal volume (V_T_) 10-12 ml kg^−1^, positive end-expiratory pressure (PEEP) of 5 cm H_2_O, respiratory rate (RR) at 12-20 breaths min^−1^ and fraction of inspired oxygen (F_I_O_2_) of 100%.

### ARDS induction

Gastric aspiration injury was induced by positioning a bronchoscope tip into bilateral mainstem bronchi to deliver 30-50 ml standardized gastric contents (pH 2) (Fraisse et al., 2007; Guenthart et al., 2019; Meers et al., 2011b). Lipopolysaccharide (LPS) from *Escherichia coli* O55:B5 was then infused centrally for 30-60 min (see Fig. 1A for experimental overview).

ARDS 0 h was defined as the time when the PaO_2_:F_I_O_2_ ratio first decreased to <150 mm Hg following LPS infusion. At ARDS 0 h, lung-protective ventilation was initiated (V_T_ 6 ml kg^−1^, PEEP and F_I_O_2_ per ARDSNet ‘Higher F_I_O_2_’ parameters) (Table S4); the RR was titrated to maintain pH>7.3. PEEP and F_I_O_2_ were gradually removed as tolerated, depending on pulse oximetry and PaO_2_. The muscle relaxant pancuronium bromide was used to paralyse animals with severe hypoxia and/or hypercarbia. Swine were moved into prone position every 6-12 h when able to tolerate position changes. ECMO was used as rescue therapy to prevent mortality prior to planned endpoint.

### Data and sample collection

Blood samples and chest radiographs were taken at pre-defined timepoints (Fig. 1). Experimental endpoints were ARDS 6 h (*n*=3) and ARDS 48 h. Animals were euthanized prior to planned experimental endpoint when their clinical status deteriorated despite maximal medical treatment, including vasopressor support, ECMO and/or attempted cardiopulmonary resuscitation. Inflammatory markers, i.e. interferon gamma (IFN-γ), interleukins (IL-1α, IL-1β, IL-2, IL-4, IL-6, IL-8, IL-10, IL-12, IL-18, interleukin receptor antagonist (IL1RN; also known as IL-1RA) and tumor necrosis factor (TNF; also known as TNF-α) were analyzed in duplicate by using the Porcine Cytokine 13-Plex Discovery Assay (Eve Technologies, Calgary, AB, Canada). Other markers, i.e. D-dimer, C-reactive protein (CRP) and ferritin, were measured by using commercially available enzyme-linked immunosorbent assays (ELISAs) according to the manufacturer's instructions (Table S5).

### Radiographic assessment of lung edema (RALE) scoring

Radiographs were blinded for evaluation of lung edema using the RALE score by two radiologists. Each radiologist independently scored the radiographs to evaluate the extent of consolidation and density of alveolar opacities (Table S6) (Warren et al., 2018).

### Histopathologic analysis of lung injury

Lung tissue samples were fixed, paraffin-embedded and sectioned. Hematoxylin and Eosin (H&E)-stained slides were reviewed blinded by a pulmonary pathologist using a previously described lung injury severity score (Table S7) (Guenthart et al., 2019). The pathologist was provided with background information regarding the study aims but slides were completely masked as to experimental group or timepoint, as described by Meyerholz and Beck (2018). Lung sections were stained for additional proteins and cell markers, i.e. for epithelial cell adhesion molecule (EPCAM), tight junction protein 1 (TJP1; also known as zonula occludens-1, ZO1), tight junction protein ZO-3 (TJP3, also known as zonula occludens-1, ZO3), surfactant protein C (SFTPC; also known as pro-surfactant protein C, pro-SPC), platelet and endothelial cell adhesion molecule 1 (PECAM1; also known as CD31) and P-selectin (SELP) (see Table S8 for antibody information).

### Statistical analysis

One-way analysis of variance with Tukey's multiple comparison post hoc tests and Student's *t*-tests were performed using Prism Version 9 (GraphPad). A value of *P*<0.05 was considered statistically significant.

## Supplementary Material

10.1242/dmm.049603_sup1 Supplementary information

## References

[DMM049603C1] Abraham, E., Carmody, A., Shenkar, R. and Arcaroli, J. (2000). Neutrophils as early immunologic effectors in hemorrhage- or endotoxemia-induced acute lung injury. *Am. J. Physiol. Lung Cell. Mol. Physiol.* 279, L1137-L1145. 10.1152/ajplung.2000.279.6.L1137 11076804

[DMM049603C2] Acute Respiratory Distress Syndrome Network, Brower, R. G., Matthay, M. A., Morris, A., Schoenfeld, D., Thompson, B. T. and Wheeler, A. (2000). Ventilation with lower tidal volumes as compared with traditional tidal volumes for acute lung injury and the acute respiratory distress syndrome. *N. Engl. J. Med.* 342, 1301-1308. 10.1056/NEJM200005043421801 10793162

[DMM049603C3] Araos, J., Alegria, L., Garcia, P., Damiani, F., Tapia, P., Soto, D., Salomon, T., Rodriguez, F., Amthauer, M., Erranz, B. et al. (2016). Extracorporeal membrane oxygenation improves survival in a novel 24-h pig model of severe acute respiratory distress syndrome. *Am. J. Transl. Res.* 8, 2826-2837. 27398166 PMC4931177

[DMM049603C4] Araos, J., Alegria, L., Garcia, A., Cruces, P., Soto, D., Erranz, B., Salomon, T., Medina, T., Garcia, P., Dubo, S. et al. (2021). Effect of positive end-expiratory pressure on lung injury and haemodynamics during experimental acute respiratory distress syndrome treated with extracorporeal membrane oxygenation and near-apnoeic ventilation. *Br. J. Anaesth.* 127, 807-814. 10.1016/j.bja.2021.07.031 34507822 PMC8449633

[DMM049603C5] ARDS Definition Task Force, Ranieri, V. M., Rubenfeld, G. D., Thompson, B. T., Ferguson, N. D., Caldwell, E., Fan, E., Camporota, L. and Slutsky, A. S. (2012). Acute respiratory distress syndrome: the Berlin definition. *JAMA* 307, 2526-2533. 10.1001/jama.2012.5669 22797452

[DMM049603C6] Ballard-Croft, C., Wang, D., Sumpter, L. R., Zhou, X. and Zwischenberger, J. B. (2012). Large-animal models of acute respiratory distress syndrome. *Ann. Thorac. Surg.* 93, 1331-1339. 10.1016/j.athoracsur.2011.06.107 22244649

[DMM049603C7] Beitler, J. R., Thompson, B. T., Baron, R. M., Bastarache, J. A., Denlinger, L. C., Esserman, L., Gong, M. N., LaVange, L. M., Lewis, R. J., Marshall, J. C. et al. (2022). Advancing precision medicine for acute respiratory distress syndrome. *Lancet Respir. Med.* 10, 107-120. 10.1016/S2213-2600(21)00157-0 34310901 PMC8302189

[DMM049603C8] Bellani, G., Laffey, J. G., Pham, T., Fan, E., Brochard, L., Esteban, A., Gattinoni, L., van Haren, F., Larsson, A., McAuley, D. F. et al. (2016). Epidemiology, patterns of care, and mortality for patients with acute respiratory distress syndrome in intensive care units in 50 countries. *JAMA* 315, 788-800. 10.1001/jama.2016.0291 26903337

[DMM049603C9] Borges, A. M., Ferrari, R. S., Thomaz, L., Ulbrich, J. M., Félix, E. A., Silvello, D. and Andrade, C. F. (2019). Challenges and perspectives in porcine model of acute lung injury using oleic acid. *Pulm. Pharmacol. Ther.* 59, 101837. 10.1016/j.pupt.2019.101837 31491506

[DMM049603C10] Cardinal-Fernandez, P., Lorente, J. A., Ballén-Barragán, A. and Matute-Bello, G. (2017). Acute respiratory distress syndrome and diffuse alveolar damage. new insights on a complex relationship. *Ann. Am. Thorac. Soc.* 14, 844-850. 10.1513/AnnalsATS.201609-728PS 28570160

[DMM049603C11] Combes, A., Hajage, D., Capellier, G., Demoule, A., Lavoue, S., Guervilly, C., Da Silva, D., Zafrani, L., Tirot, P., Veber, B. et al. (2018). Extracorporeal membrane oxygenation for severe acute respiratory distress syndrome. *N. Engl. J. Med.* 378, 1965-1975. 10.1056/NEJMoa1800385 29791822

[DMM049603C12] Folkesson, H. G., Matthay, M. A., Hébert, C. A. and Broaddus, V. C. (1995). Acid aspiration-induced lung injury in rabbits is mediated by interleukin-8-dependent mechanisms. *J. Clin. Invest.* 96, 107-116. 10.1172/JCI118009 7615779 PMC185178

[DMM049603C13] Fraisse, A., Bregeon, F., Delpierre, S., Gaudart, J., Payan, M. J., Pugin, J. and Papazian, L. (2007). Hemodynamics in experimental gastric juice induced aspiration pneumonitis. *Intensive Care Med.* 33, 300-307. 10.1007/s00134-006-0457-2 17160420

[DMM049603C14] Fuchs, L., Feng, M., Novack, V., Lee, J., Taylor, J., Scott, D., Howell, M., Celi, L. and Talmor, D. (2019). The effect of ARDS on survival: do patients die from ARDS or with ARDS? *J. Intensive Care Med.* 34, 374-382. 10.1177/0885066617717659 28681644

[DMM049603C15] Grommes, J. and Soehnlein, O. (2011). Contribution of neutrophils to acute lung injury. *Mol. Med.* 17, 293-307. 10.2119/molmed.2010.00138 21046059 PMC3060975

[DMM049603C16] Guenthart, B. A., O'Neill, J. D., Kim, J., Queen, D., Chicotka, S., Fung, K., Simpson, M., Donocoff, R., Salna, M., Marboe, C. C. et al. (2019). Regeneration of severely damaged lungs using an interventional cross-circulation platform. *Nat. Commun.* 10, 1985. 10.1038/s41467-019-09908-1 31064987 PMC6504972

[DMM049603C17] Lee, J. W., Fang, X., Gupta, N., Serikov, V. and Matthay, M. A. (2009). Allogeneic human mesenchymal stem cells for treatment of E. coli endotoxin-induced acute lung injury in the ex vivo perfused human lung. *Proc. Natl. Acad. Sci. USA* 106, 16357-16362. 10.1073/pnas.0907996106 19721001 PMC2735560

[DMM049603C18] Leiphrakpam, P. D., Weber, H. R., McCain, A., Matas, R. R., Duarte, E. M. and Buesing, K. L. (2021). A novel large animal model of smoke inhalation-induced acute respiratory distress syndrome. *Respir. Res.* 22, 198. 10.1186/s12931-021-01788-8 34233680 PMC8261975

[DMM049603C19] Matthay, M. A., McAuley, D. F. and Ware, L. B. (2017). Clinical trials in acute respiratory distress syndrome: challenges and opportunities. *Lancet Respir. Med.* 5, 524-534. 10.1016/S2213-2600(17)30188-1 28664851

[DMM049603C20] Matthay, M. A., Calfee, C. S., Zhuo, H., Thompson, B. T., Wilson, J. G., Levitt, J. E., Rogers, A. J., Gotts, J. E., Wiener-Kronish, J. P., Bajwa, E. K. et al. (2019). Treatment with allogeneic mesenchymal stromal cells for moderate to severe acute respiratory distress syndrome (START study): a randomised phase 2a safety trial. *Lancet Respir. Med.* 7, 154-162. 10.1016/S2213-2600(18)30418-1 30455077 PMC7597675

[DMM049603C21] Matute-Bello, G., Frevert, C. W. and Martin, T. R. (2008). Animal models of acute lung injury. *Am. J. Physiol. Lung Cell. Mol. Physiol.* 295, L379-L399. 10.1152/ajplung.00010.2008 18621912 PMC2536793

[DMM049603C22] Matute-Bello, G., Downey, G., Moore, B. B., Groshong, S. D., Matthay, M. A., Slutsky, A. S. and Kuebler, W. M. and Acute Lung Injury in Animals Study Group. (2011). An official American Thoracic Society workshop report: features and measurements of experimental acute lung injury in animals. *Am. J. Respir. Cell Mol. Biol.* 44, 725-738. 10.1165/rcmb.2009-0210ST 21531958 PMC7328339

[DMM049603C23] Meduri, G. U., Headley, S., Kohler, G., Stentz, F., Tolley, E., Umberger, R. and Leeper, K. (1995). Persistent elevation of inflammatory cytokines predicts a poor outcome in ARDS. Plasma IL-1β and IL-6 levels are consistent and efficient predictors of outcome over time. *Chest* 107, 1062-1073. 10.1378/chest.107.4.1062 7705118

[DMM049603C24] Meers, C. M., De Wever, W., Verbeken, E., Mertens, V., Wauters, S., De Vleeschauwer, S. I., Vos, R., Vanaudenaerde, B. M., Verleden, G. M. and Van Raemdonck, D. E. (2011a). A porcine model of acute lung injury by instillation of gastric fluid. *J. Surg. Res.* 166, e195-e204. 10.1016/j.jss.2010.10.015 21109258

[DMM049603C25] Meers, C. M., Tsagkaropoulos, S., Wauters, S., Verbeken, E., Vanaudenaerde, B., Scheers, H., Verleden, G. M. and Van Raemdonck, D. (2011b). A model of ex vivo perfusion of porcine donor lungs injured by gastric aspiration: a step towards pretransplant reconditioning. *J. Surg. Res.* 170, e159-e167. 10.1016/j.jss.2011.05.015 21737098

[DMM049603C26] Meyerholz, D. K. and Beck, A. P. (2018). Principles and approaches for reproducible scoring of tissue stains in research. *Lab. Invest.* 98, 844-855. 10.1038/s41374-018-0057-0 29849125

[DMM049603C27] Millar, J. E., Bartnikowski, N., Passmore, M. R., Obonyo, N. G., Malfertheiner, M. V., von Bahr, V., Redd, M. A., See Hoe, L., Ki, K. K., Pedersen, S. et al. (2020). Combined mesenchymal stromal cell therapy and extracorporeal membrane oxygenation in acute respiratory distress syndrome. A randomized controlled trial in sheep. *Am. J. Respir. Crit. Care. Med.* 202, 383-392. 10.1164/rccm.201911-2143OC 32293914 PMC7397785

[DMM049603C28] National Research Council. (2011). *Guide for the Care and Use of Laboratory Animals*, 8th edn. Washington, DC: The National Academies Press.

[DMM049603C29] Nieman, G. F., Gatto, L. A., Paskanik, A. M., Yang, B., Fluck, R. and Picone, A. (1996). Surfactant replacement in the treatment of sepsis-induced adult respiratory distress syndrome in pigs. *Crit. Care Med.* 24, 1025-1033. 10.1097/00003246-199606000-00024 8681569

[DMM049603C41] O’Neill, J., Guenthart, B., Kim, J., Chicotka, S., Queen, D., Fung, K., Marboe, C., Romanov, A., Huang, S. X. L., Chen, Y.-W. et al. (2017). Cross-circulation for extracorporeal support and recovery of the lung. *Nat. Biomed. Eng.* 1, 0037. 10.1038/s41551-017-0037

[DMM049603C30] Papazian, L., Forel, J. M., Gacouin, A., Penot-Ragon, C., Perrin, G., Loundou, A., Jaber, S., Arnal, J. M., Perez, D., Seghboyan, J. M. et al. (2010). Neuromuscular blockers in early acute respiratory distress syndrome. *N. Engl. J. Med.* 363, 1107-1116. 10.1056/NEJMoa1005372 20843245

[DMM049603C31] Peek, G. J., Mugford, M., Tiruvoipati, R., Wilson, A., Allen, E., Thalanany, M. M., Hibbert, C. L., Truesdale, A., Clemens, F., Cooper, N. et al. (2009). Efficacy and economic assessment of conventional ventilatory support versus extracorporeal membrane oxygenation for severe adult respiratory failure (CESAR): a multicentre randomised controlled trial. *Lancet* 374, 1351-1363. 10.1016/S0140-6736(09)61069-2 19762075

[DMM049603C32] Puneet, P., Moochhala, S. and Bhatia, M. (2005). Chemokines in acute respiratory distress syndrome. *Am. J. Physiol. Lung Cell. Mol. Physiol.* 288, L3-L15. 10.1152/ajplung.00405.2003 15591040 PMC7191630

[DMM049603C33] Schütte, H., Lohmeyer, J., Rosseau, S., Ziegler, S., Siebert, C., Kielisch, H., Pralle, H., Grimminger, F., Morr, H. and Seeger, W. (1996). Bronchoalveolar and systemic cytokine profiles in patients with ARDS, severe pneumonia and cardiogenic pulmonary oedema. *Eur. Respir. J.* 9, 1858-1867. 10.1183/09031936.96.09091858 8880103

[DMM049603C34] Semler, M. W., Bernard, G. R., Aaron, S. D., Angus, D. C., Biros, M. H., Brower, R. G., Calfee, C. S., Colantuoni, E. A., Ferguson, N. D., Gong, M. N. et al. (2020). Identifying clinical research priorities in adult pulmonary and critical care. NHLBI working group report. *Am. J. Respir. Crit. Care. Med.* 202, 511-523. 10.1164/rccm.201908-1595WS 32150460 PMC7427373

[DMM049603C35] Shaw, T. D., McAuley, D. F. and O'Kane, C. M. (2019). Emerging drugs for treating the acute respiratory distress syndrome. *Expert Opin Emerg. Drugs* 24, 29-41. 10.1080/14728214.2019.1591369 30841764

[DMM049603C36] Tiba, M. H., McCracken, B. M., Leander, D. C., Colmenero, C. I., Nemzek, J. A., Sjoding, M. W., Konopka, K. E., Flott, T. L., VanEpps, J. S., Daniels, R. C. et al. (2021). A novel swine model of the acute respiratory distress syndrome using clinically relevant injury exposures. *Physiol. Rep.* 9, e14871. 10.14814/phy2.14871 33991456 PMC8123544

[DMM049603C37] Warren, M. A., Zhao, Z., Koyama, T., Bastarache, J. A., Shaver, C. M., Semler, M. W., Rice, T. W., Matthay, M. A., Calfee, C. S. and Ware, L. B. (2018). Severity scoring of lung oedema on the chest radiograph is associated with clinical outcomes in ARDS. *Thorax* 73, 840-846. 10.1136/thoraxjnl-2017-211280 29903755 PMC6410734

[DMM049603C38] Whitney, J. E., Zhang, B., Koterba, N., Chen, F., Bush, J., Graham, K., Lacey, S. F., Melenhorst, J. J., Teachey, D. T., Mensinger, J. L. et al. (2020). Systemic endothelial activation is associated with early acute respiratory distress syndrome in children with extrapulmonary sepsis. *Crit. Care Med.* 48, 344-352. 10.1097/CCM.0000000000004091 32058372 PMC8749338

[DMM049603C39] Williams, A. E. and Chambers, R. C. (2014). The mercurial nature of neutrophils: still an enigma in ARDS? *Am. J. Physiol. Lung Cell. Mol. Physiol.* 306, L217-L230. 10.1152/ajplung.00311.2013 24318116 PMC3920201

[DMM049603C40] Yadav, H., Bartley, A., Keating, S., Meade, L. A., Norris, P. J., Carter, R. E., Gajic, O. and Kor, D. J. (2018). Evolution of validated biomarkers and intraoperative parameters in the development of postoperative ARDS. *Respir. Care* 63, 1331-1340. 10.4187/respcare.06103 29921605

