# Supplementary material for: A clinically relevant model of acute respiratory distress syndrome in human-size swine
Source: Dis Model Mech. 2022 Oct 10;15(10):dmm049603. doi: 10.1242/dmm.049603 (PMC9586570; doi:10.1242/dmm.049603)
Supplement: Supplementary information [file dmm-15-049603-s1.pdf]

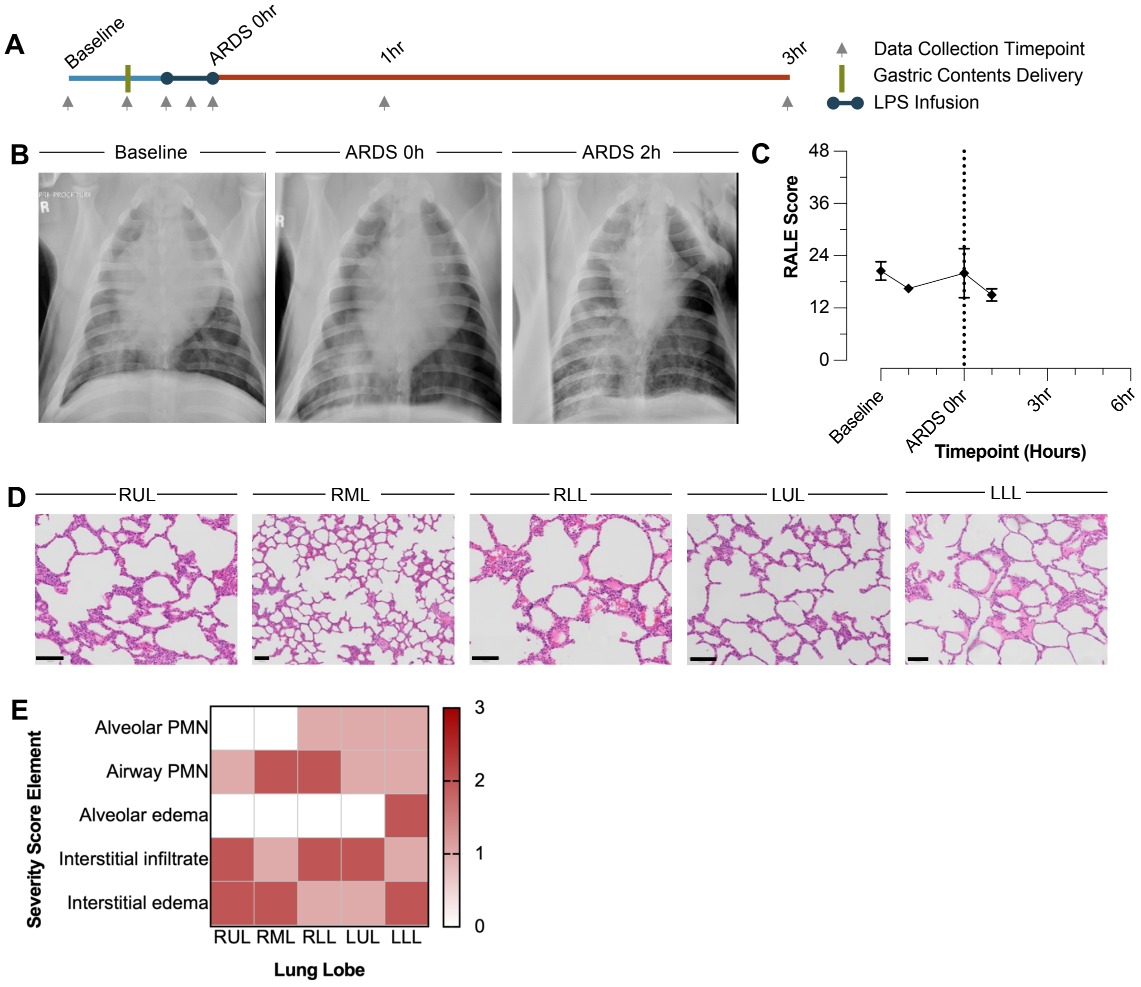

Fig. S1. ARDS 1 - 54411. (A) Experimental timecourse. (B) Chest radiographs at baseline, ARDS 0hr, and ARDS 2hr. (C) Radioagraphic Assessment of Lung Edema scores. (D) H&E section from lung tissue at study endpoint. Scale bar = 100 μm. (E) Heatmap of lung injury severity score by score element and lung lobe. GC = gastric contents, LPS = lipopolysaccharide, RALE = Radiographic Assessment of Lung Edema, RUL = right upper lobe, RML = right middle lobe, RLL = right lower lobe, LUL = left upper lobe, LLL = left lower lobe.

Disease Models & Mechanisms • Supplementary information

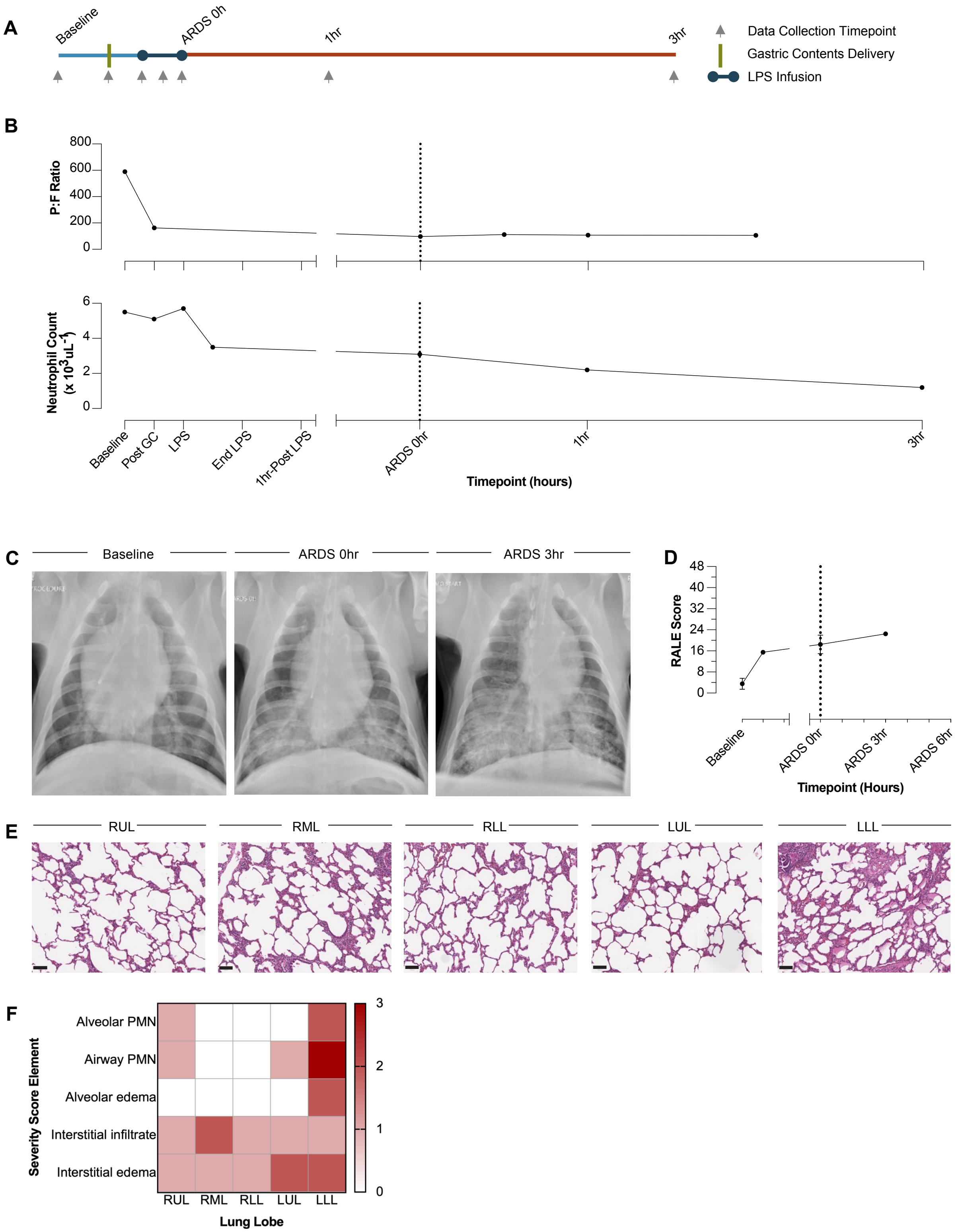

Fig. S2. ARDS 9 - 55369. (A) Experimental timecourse. (B) Pa_{O2}:FI_{O2} ratio over experimental timecourse; neutrophil count (x10^3µL^{-1}). (C) Chest radiographs at baseline, ARDS 0hr, and ARDS 3hr. (D) Radioagraphic Assessment of Lung Edema scores. (E) H&E section from lung tissue at study endpoint. Scale bar = 100 µm. (F) Heatmap of lung injury severity score by score element and lung lobe. GC = gastric contents, LPS = lipopolysaccharide, RALE = Radiographic Assessment of Lung Edema, RUL = right upper lobe, RML = right middle lobe, RLL = right lower lobe, LUL = left upper lobe, LLL = left lower lobe.

Disease Models & Mechanisms • Supplementary information

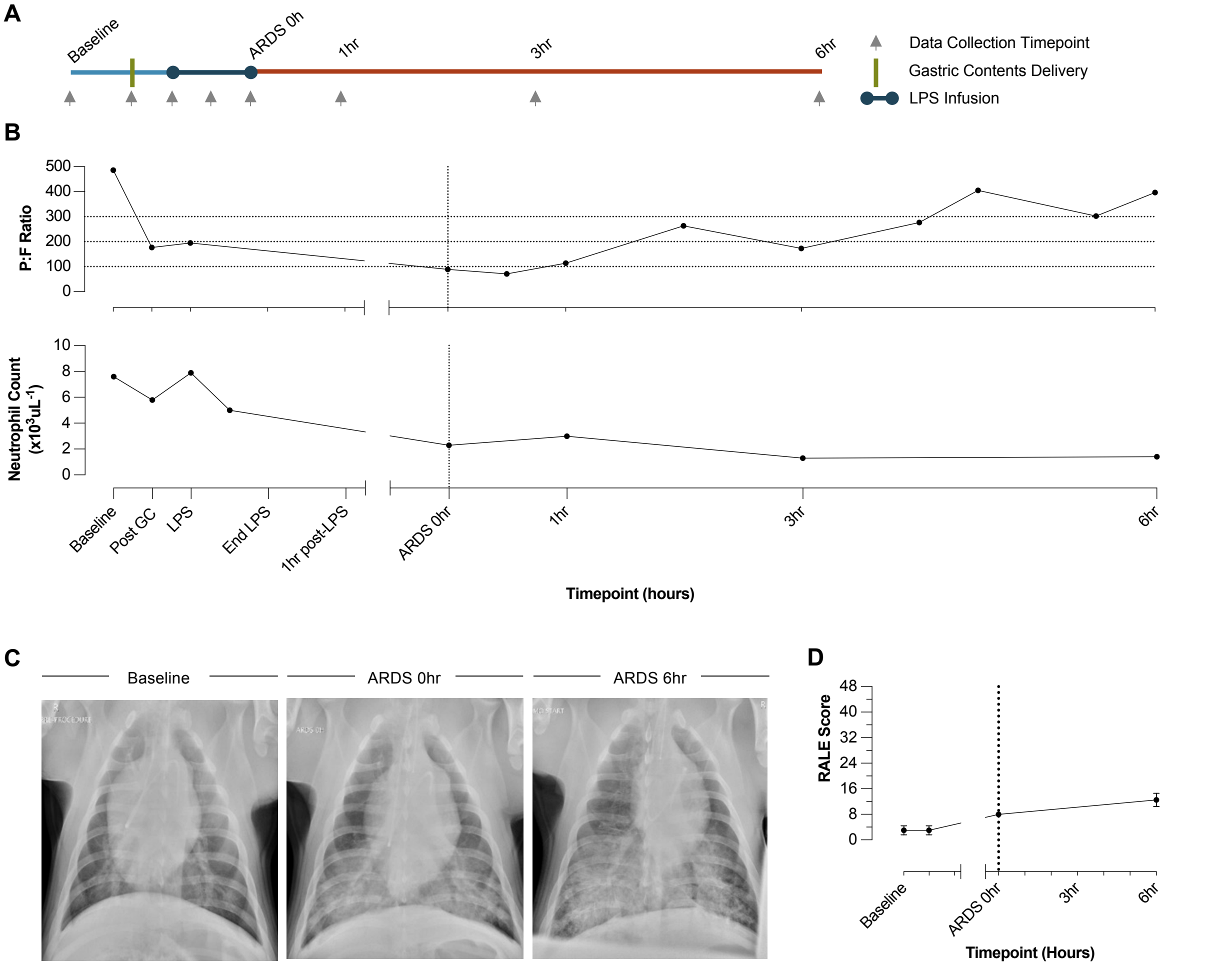

Fig. S3. ARDS 6 - 54990. (A) Experimental timecourse. (B) Pa_{O2}:FI_{O2} ratio over experimental timecourse; neutrophil count ($x10^3 \mu L^{-1}$). (C) Chest radiographs at baseline, ARDS 0hr, and ARDS 6hr. (D) Radioagraphic Assessment of Lung Edema scores. GC = gastric contents, LPS = lipopolysaccharide, RALE = Radiographic Assessment of Lung Edema, RUL = right upper lobe, RML = right middle lobe, RLL = right lower lobe, LUL = left upper lobe, LLL = left lower lobe.

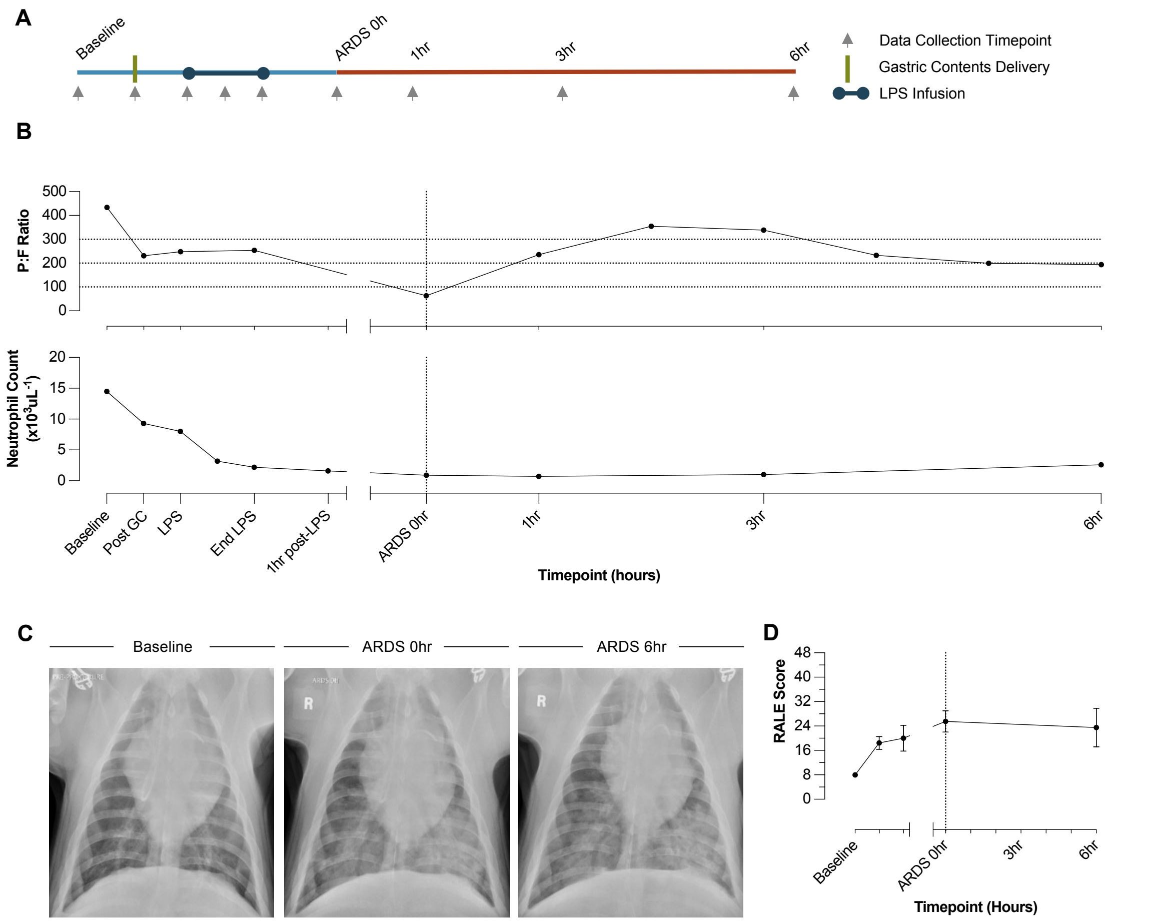

Fig. S4. ARDS 8 - 55214. (A) Experimental timecourse. (B) Pa_{O2}:FI_{O2} ratio over experimental timecourse; neutrophil count ($x10^3 \mu L^{-1}$). (C) Chest radiographs at baseline, ARDS 0hr, and ARDS 6hr. (D) Radioagraphic Assessment of Lung Edema scores. GC = gastric contents, LPS = lipopolysaccharide, RALE = Radiographic Assessment of Lung Edema, RUL = right upper lobe, RML = right middle lobe, RLL = right lower lobe, LUL = left upper lobe, LLL = left lower lobe.

Disease Models & Mechanisms • Supplementary information

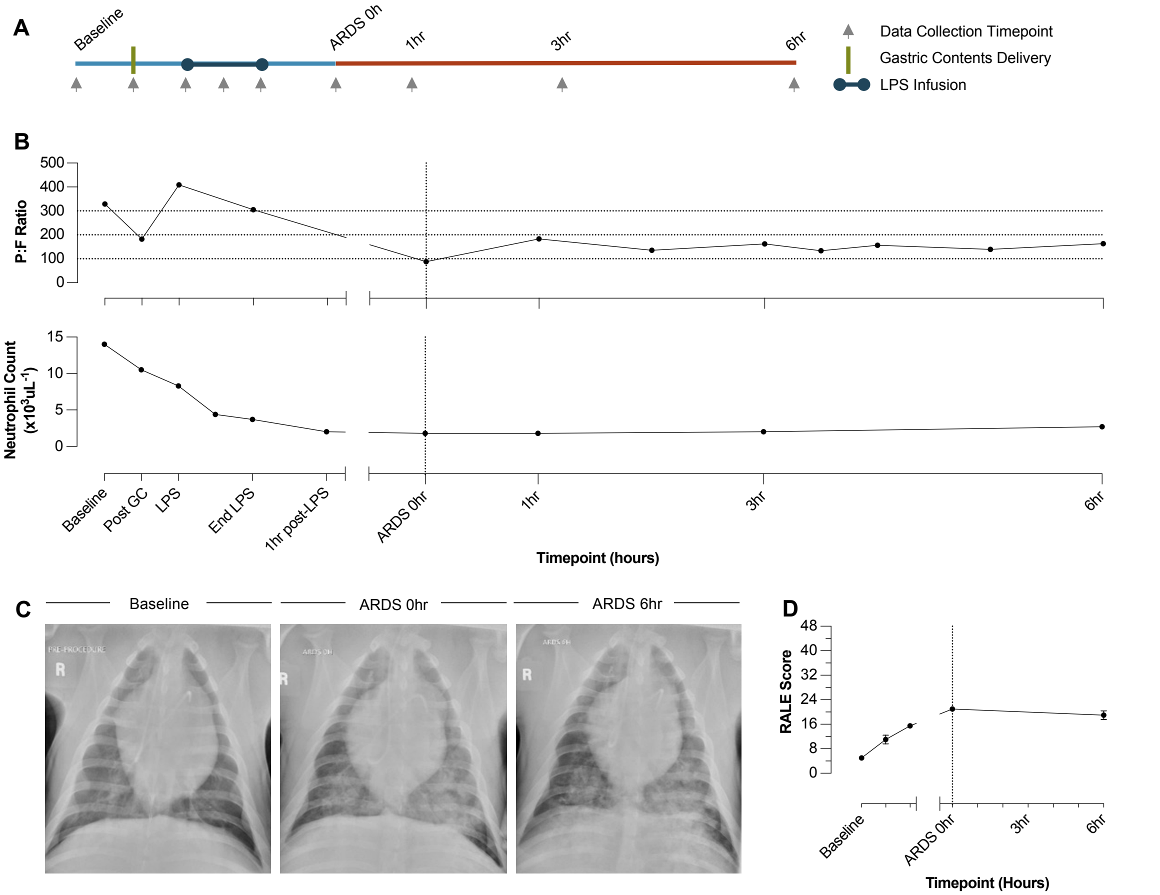

Fig. S5. ARDS 10 - 55368. (A) Experimental timecourse. (B) $Pa_{O2}:FI_{O2}$ ratio over experimental timecourse; neutrophil count ($x10^3 \mu L^{-1}$). (C) Chest radiographs at baseline, ARDS 0hr, and ARDS 6hr. (D) Radioagraphic Assessment of Lung Edema scores. GC = gastric contents, LPS = lipopolysaccharide, RALE = Radiographic Assessment of Lung Edema, RUL = right upper lobe, RML = right middle lobe, RLL = right lower lobe, LUL = left upper lobe, LLL = left lower lobe.

Disease Models & Mechanisms • Supplementary information

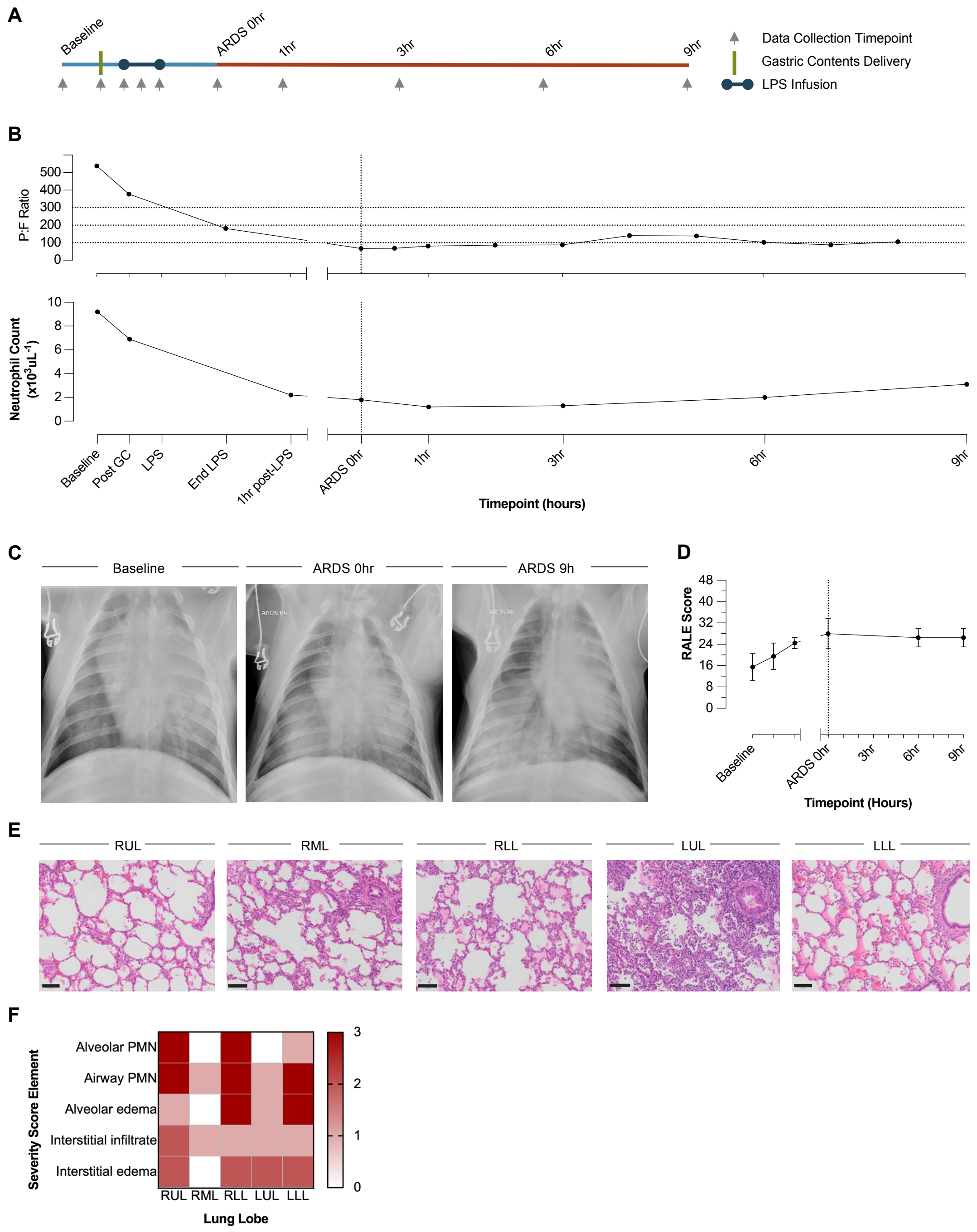

Fig. S6. ARDS 3 - 54633. (A) Experimental timecourse. (B) Pa_{O2}:FI_{O2} ratio over experimental timecourse; neutrophil count ($x10^3 \mu L^{-1}$). (C) Chest radiographs at baseline, ARDS 0hr, and ARDS 9hr. (D) Radioagraphic Assessment of Lung Edema scores. (E) H&E section from lung tissue at study endpoint. Scale bar = 100 μm. (F) Heatmap of lung injury severity score by score element and lung lobe. GC = gastric contents, LPS = lipopolysaccharide, RALE = Radiographic Assessment of Lung Edema, RUL = right upper lobe, RML = right middle lobe, RLL = right lower lobe, LUL = left upper lobe, LLL = left lower lobe.

Disease Models & Mechanisms • Supplementary information

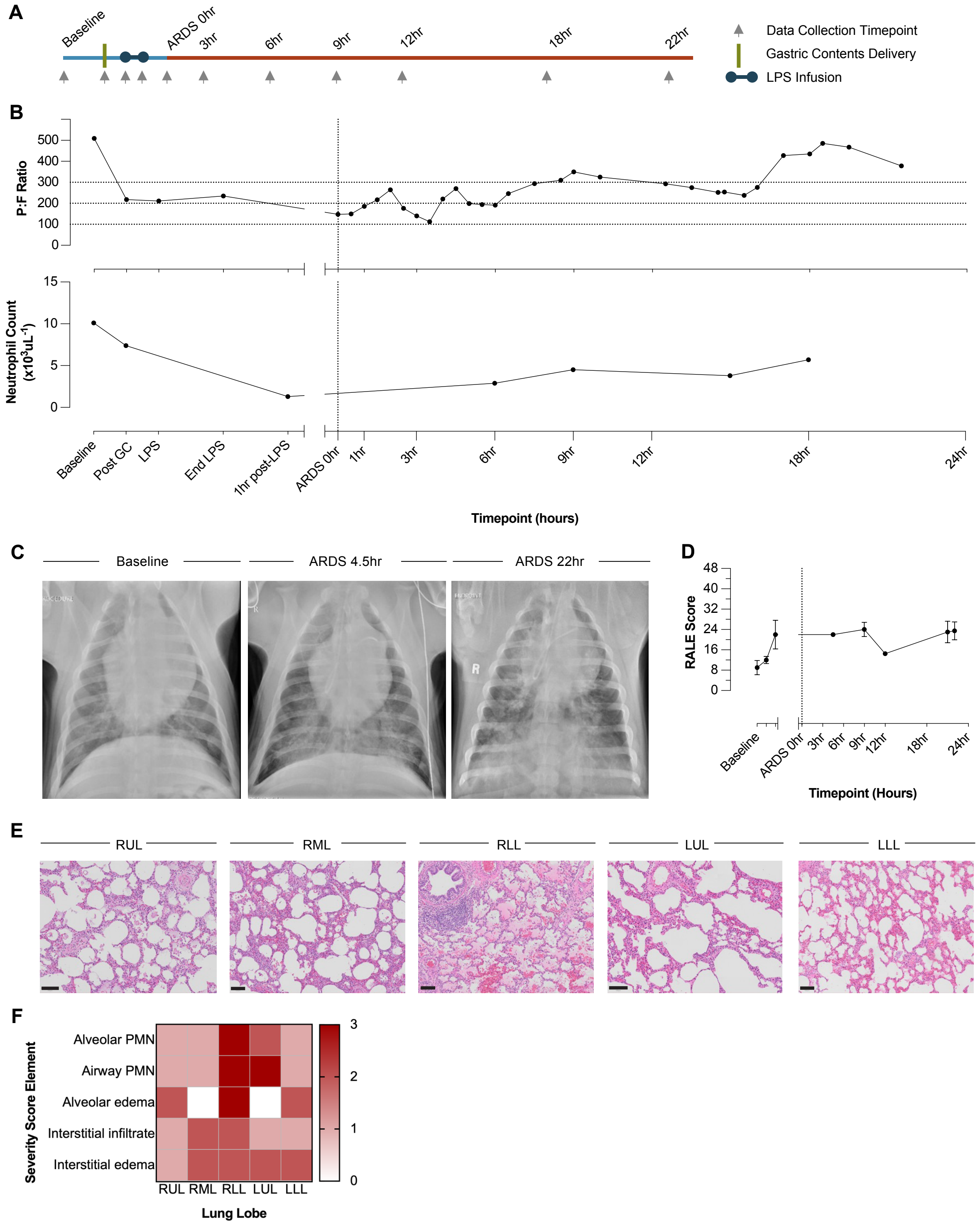

Fig. S7. ARDS 7 - 55146. (A) Experimental timecourse. (B) Pa_{O2}:FI_{O2} ratio over experimental timecourse; neutrophil count ($x10^3 \mu L^{-1}$). (C) Chest radiographs at baseline, ARDS 4.5hr, and ARDS 22hr. (D) Radioagraphic Assessment of Lung Edema scores. (E) H&E section from lung tissue at study endpoint. Scale bar = 100 µm. (F) Heatmap of lung injury severity score by score element and lung lobe. GC = gastric contents, LPS = lipopolysaccharide, RALE = Radiographic Assessment of Lung Edema, RUL = right upper lobe, RML = right middle lobe, RLL = right lower lobe, LUL = left upper lobe, LLL = left lower lobe.

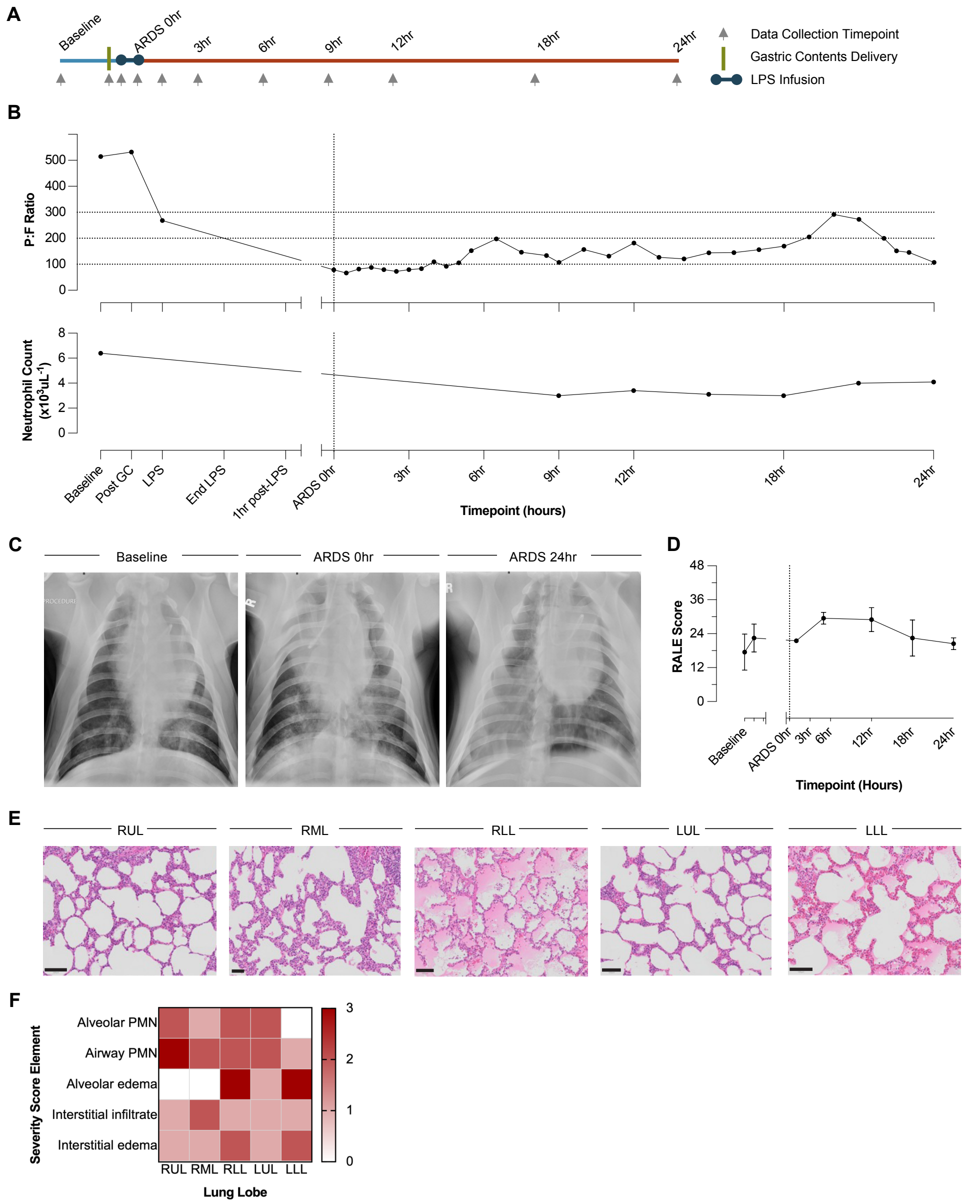

Fig. S8. ARDS 2 - 54456. (A) Experimental timecourse. (B) Pa_{O2}:FI_{O2} ratio over experimental timecourse; neutrophil count (x10^3μL^{-1}). (C) Chest radiographs at baseline, ARDS 0hr, and ARDS 24hr. (D) Radioagraphic Assessment of Lung Edema scores. (E) H&E section from lung tissue at study endpoint. Scale bar = 100 μm. (F) Heatmap of lung injury severity score by score element and lung lobe. GC = gastric contents, LPS = lipopolysaccharide, RALE = Radiographic Assessment of Lung Edema, RUL = right upper lobe, RML = right middle lobe, RLL = right lower lobe, LUL = left upper lobe, LLL = left lower lobe.

Disease Models & Mechanisms • Supplementary information

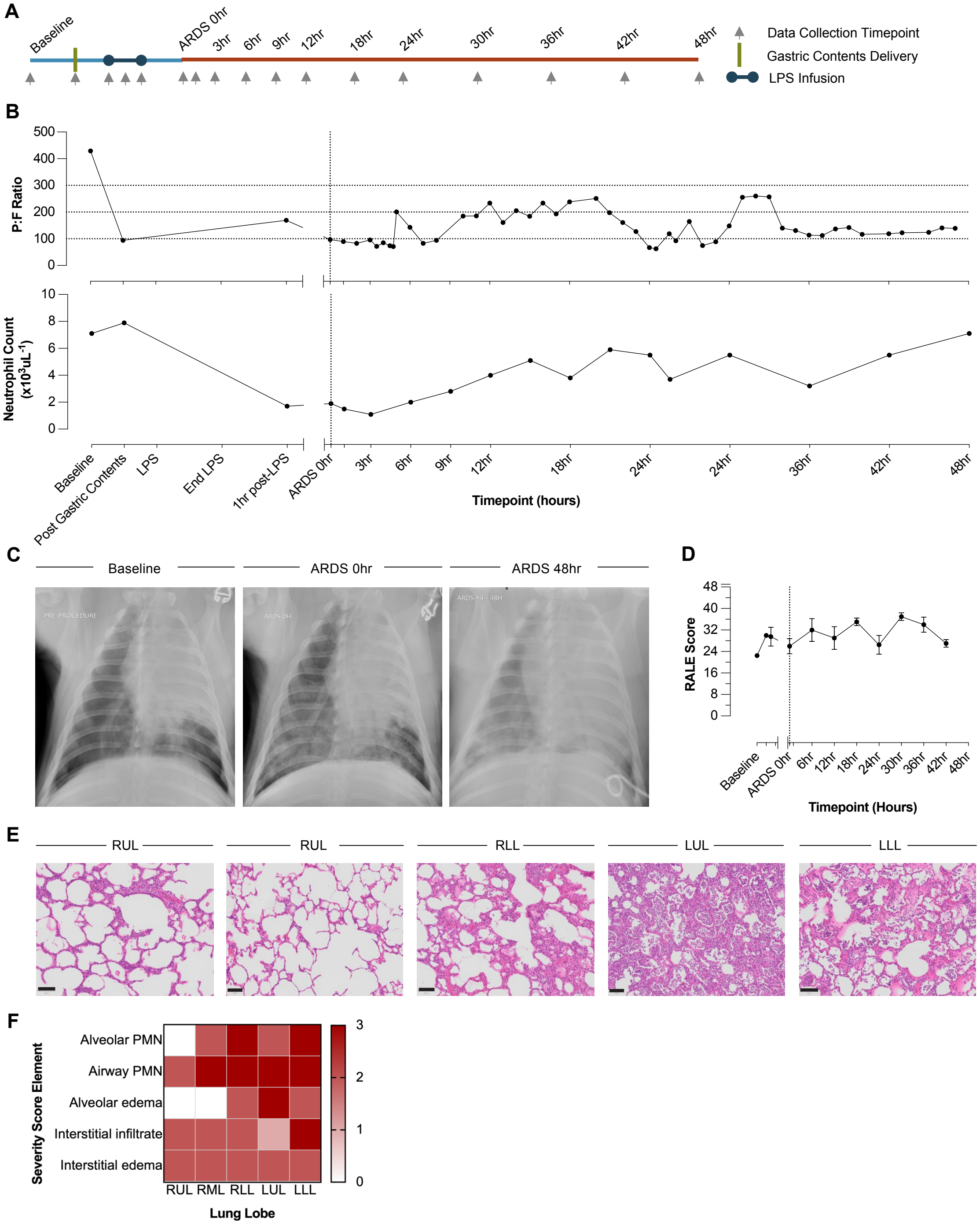

Fig. S9. ARDS 4 - 54659. (A) Experimental timecourse. (B) Pa_{O2}:FI_{O2} ratio over experimental timecourse; neutrophil count (x10^3µL^{-1}). (C) Chest radiographs at baseline, ARDS 0hr, and ARDS 48hr. (D) Radioagraphic Assessment of Lung Edema scores. (E) H&E section from lung tissue at study endpoint. Scale bar = 100 µm. (F) Heatmap of lung injury severity score by score element and lung lobe. GC = gastric contents, LPS = lipopolysaccharide, RALE = Radiographic Assessment of Lung Edema, RUL = right upper lobe, RML = right middle lobe, RLL = right lower lobe, LUL = left upper lobe, LLL = left lower lobe.

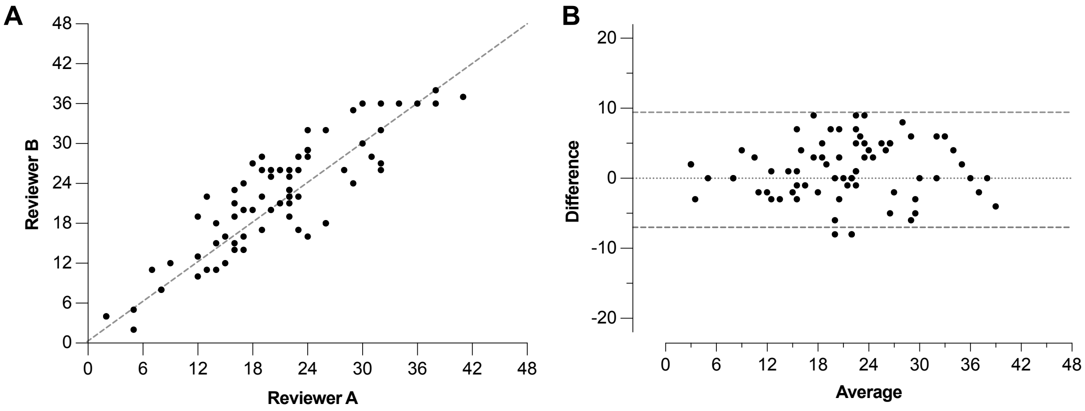

Fig. S10. Agreement on RALE scoring between two independent radiologists. (A) Correlation plot. (B) Bland-Altman plot with average score between two reviewers and difference in score; 95% limits of agreement shown in dashed grey lines. RALE = Radiographic Assessment of Lung Edema scoring

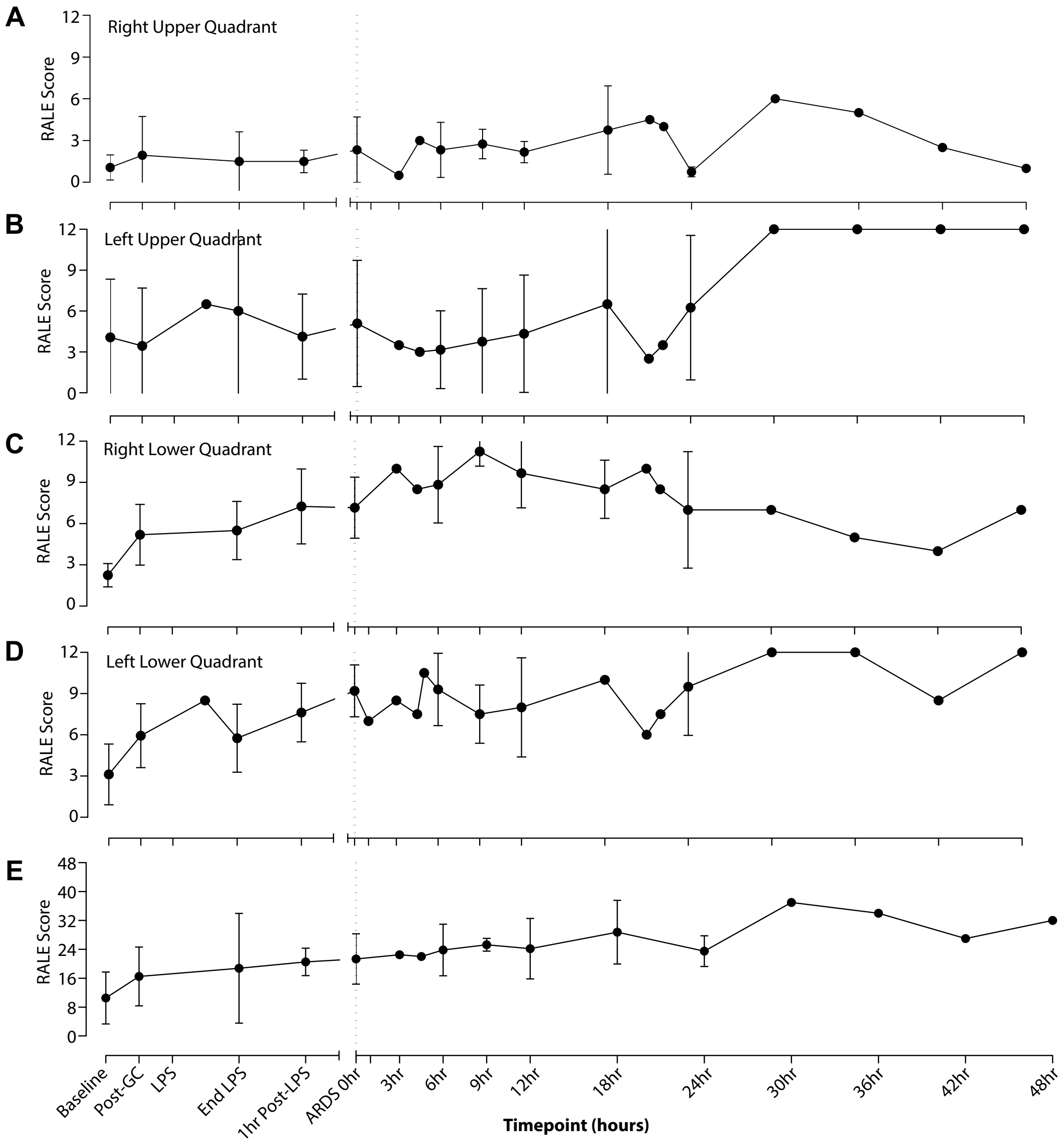

Fig. S11. Radiographic Assessment of Lung Edema scoring from Warren et al. Severity scoring of lung oedema on the chest radiograph is associated with clinical outcomes. Thorax. 2018;73:840-846. (A) RALE score for Right Upper Quadrant; (B) RALE score for Left Upper Quadrant ; (C) RALE score for Right Lower Quadrant ; (D) RALE score for Left Lower Quadrant ; (E) Total RALE Score. GC = Gastric Contents, LPS = Lipopolysaccharides, RALE = Radiographic Assessment of Lung Edema scoring

Table S1. Berlin Criteria from ARDS Definition Task Force, Ranieri VM, Rubenfeld GD, et al. Acute respiratory distress syndrome: the Berlin Definition. JAMA. 2012;307(23):2526-2533.

**Timing**	Within 1 week of a known clinical insult or new or worsening respiratory symptoms
**Chest imaging** (CXR or CT)	Bilateral opacities – not fully explained by effusions, lobar/lung collapse, or nodules
**Origin of edema**	Respiratory failure not fully explained by cardiac failure or fluid overload<br>Need objective assessment (e.g. echocardiography) to exclude hydrostatic edema if no risk factor present
**Oxygenation***	
Mild	200 mm Hg < PaO_2/F_IO_2 ≤ 300 mm Hg
Moderate	100 mm Hg < PaO_2/F_IO_2 ≤ 200 mm Hg
Severe	PaO_2/F_IO_2 ≤ 100 mm Hg

*with PEEP ≥5 cm H_2O

Table S2. Baseline measurements of animals

Animal Number	Sex	Age	Weight (kg)	P:F	Arterial Blood Pressure	Pulmonary Artery Pressure	CXR RALE Score
54411	Male	Adult	60.9	516	105/40	Not collected	20.5
54456	Female	Adult	55.4	514	97/60	33/17	17.5
54633	Female	Adult	47	539	106/70	28/17	15.5
54659	Female	Adult	48	429	97/52	36/24	22.5
54990	Female	Adult	45.6	462	122/75	18/10	3
55146	Female	Adult	45	510	100/68	22/12	9
55214	Female	Adult	50	498	104/57	20/12	8
55368	Female	Adult	45	448	104/58	29/15	21
55369	Female	Adult	46.4	543	106/72	19/13	3.5

Table S3. ARDS induction timeline

	Gastric Contents to LPS	LPS Infusion Time	LPS Infusion End to ARDS 0hr
54411	31 min	1 hr	0 hr
54456	3 min	0.5 hr	0 hr
54633	10 min	1 hr	1.5 hr
54659	20 min	1 hr	2.75 hr
54990	20 min	1 hr	0 hr
55146	30 min	1 hr	2.5 hr
55214	35 min	1 hr	4 hr
55369	30 min	1 hr	0 hr
55368	90 min	1 hr	2 hr

Disease Models & Mechanisms • Supplementary information

Table S4. Higher PEEP/lower FIO2 ARDSNet ventilator parameters from The Acute Respiratory Distress Syndrome Network. Ventilation with lower tidal volumes as compared with traditional tidal volumes for acute lung injury and the acute respiratory distress syndrome. ***N Engl J Med***. 2000;342(18):1301-1308.

F_IO_2	0.3	0.3	0.3	0.3	0.3	0.4	0.4	0.5	0.5	0.5-0.8	0.8	0.9	1.0	1.0
PEEP (mm H_2O)	5	8	10	12	14	14	16	16	18	20	22	22	22	24

Table S5. ELISA information

**ELISA**	**Manufacturer**	**Catalogue Number**	**Sample Type**	**Dilution Factor**
C Reactive Protein	Abcam	ab205089	Plasma	1:2000
D-Dimer	LS Bio	LS-F56441	Plasma	1:20
Ferritin	Novus Biologicals	NBP2-7537-1	Serum	1:100

Table S6. Radiographic Assessment of Lung Edema scoring from Warren ***et al***. Severity scoring of lung oedema on the chest radiograph is associated with clinical outcomes. ***Thorax***. 2018;73:840-846.

		**Consolidation Score**			
Score	0	1	2	3	4
Extent of alveolar opacities	None	<25%	25 – 50%	50 – 75%	> 75%

	**Density Score:**		
Score	1	2	3
Density of alveolar opacities	Hazy	Moderate	Dense

Quadrant Score = Consolidation Score x Density Score
Total Score = Q1 + Q2 + Q3 + Q4

Disease Models & Mechanisms • Supplementary information

Table S7. Histopathologic lung injury severity score from Guenthart BA, O'Neill JD, Kim J, et al. Regeneration of severely damaged lungs using an interventional cross-circulation platform. *Nat Commun*. 2019;10(1):1985.

**Airway PMN / hpf**				
Score	0	1	2	3
Bronchi and bronchioles (%) containing any neutrophils	0	1-25	26-50	> 50
**Alveolar PMN / hpf**				
Score	0	1	2	3
Alveoli (%) more than half-filled with neutrophils	0	1-25	26-50	> 50
**Alveolar edema**				
Score	0	1	2	3
Alveoli (%) with edema	< 5	6-25	26-50	> 50
**Interstitial edema**				
Score	0	1	2	
Perivascular and peribronchial spaces expanded with edematous fluid	< 5	1x width vessel media	≥ 2x width vessel media	
**Interstitial infiltrate / hpf**				
Score	0	1	2	3
Lymphocytes/neutrophils in interstitium around vessels and airways and in alveolar septa and pleura	0	< 50	50-100	> 100

Disease Models & Mechanisms • Supplementary information

Table S8. Antibodies

PRIMARY ANTIBODIES				
**Antibody**	**Manufacturer**	**Catalogue Number**	**Host**	**Dilution**
CD31	Abcam	ab28364	Rabbit	1/50
EpCAM	Abcam	ab71916	Rabbit	1/100
pro-Surfactant Protein C	Abcam	ab90716	Rabbit	1/100
P-Selectin	Abcam	ab202983	Rabbit	1/200
Zonula Occludens-1	Abcam	ab190085	Goat	1/100
Zonula Occludens-3	Abcam	ab205882	Rabbit	1/250
SECONDARY ANTIBODIES				
**Fluorophore**	**Manufacturer**	**Catalogue Number**	**Host / Target**	**Dilution**
Alexa Fluor 488	Invitrogen	A-21206	Donkey / Rabbit	1/200
Alexa Fluor 488	Invitrogen	A-21202	Donkey / Mouse	1/200
Alexa Fluor 555	Invitrogen	A-31572	Donkey / Rabbit	1/200
Alexa Fluor 555	Invitrogen	A-31571	Donkey / Mouse	1/200
Alexa Fluor 488	Invitrogen	A-11055	Donkey / Goat	1/200
PRIMARY CONJUGATION KITS				
FITC Conjugation Kit	Abcam	ab188285		
AF555 Conjugation Kit	Abcam	ab269820		
AF647 Conjugation Kit	Abcam	ab269823		

Disease Models & Mechanisms • Supplementary information

Supplementary Materials and Methods

Preprocedure preparation and hemodynamic monitoring

Swine were vaccinated from the vendor for porcine reproductive and respiratory syndrome, *Mycoplasma hyopneumoniae*, *Haemophilus parasuis*, Swine Influenza (H1N1, H3N2), parvovirus, leptospirosis, *Erysipelas spp.*, *E. coli*, *Pasteurella pneumotropica*, and *Bordetella bronchiseptica*. Five days prior to shipment, animals were treated with oxytetracycline (8lb/ton) in food. Animals were housed at Columbia in an AAALAC-accredited facility. All conducted studies were approved by the IACUC at Columbia University.

Swine underwent general anesthesia via intramuscular induction with tiletamine/zolazepam (5 mg kg^{-1}, Zoetis). A 10Fr urinary catheter was placed into the urethra or percutaneously into the bladder, if necessary, to monitor urine output. Peripheral and central venous catheters and arterial catheters were placed percutaneously under ultrasound guidance. A 5Fr introducer (Cook Medical) was placed in the right external jugular vein to establish central venous access for subsequent ECMO cannulation if needed. An 8.5Fr introducer sheath (Arrow) was placed in the left external jugular vein and a 7Fr pulmonary arterial catheter (Edwards) was floated into the pulmonary artery. Placement was confirmed by chest radiograph. A 7Fr dual- or triple-lumen catheter (Arrow) was also placed in the left external jugular vein for medication infusion. A 20G arterial micropuncture kit (Cook Medical) was used to access the femoral artery and subsequently upsized to a 6-7Fr 20cm introducer sheath for continuous arterial blood pressure monitoring, and to establish arterial access for Veno-Arterial (VA)-ECMO, if required. Continuous infusion with vasopressors (norepinephrine 0.125-1.0 mcg kg^{-1} hr^{-1}, phenylephrine 1 mcg kg^{-1} min^{-1}, and dopamine 4-6 mcg kg^{-1} min^{-1}) via central venous catheter was initiated and continued as needed to maintain mean arterial blood pressure ≥ 55 mmHg.

Swine were transitioned from isoflurane anesthesia to total intravenous anesthesia utilizing propofol (1-4 mg kg^{-1} hr^{-1} or bolus as needed, Zoetis), fentanyl (3-5 mcg kg^{-1} hr^{-1}, West-Ward), dexmedetomidine (1-5 mcg kg^{-1} hr^{-1} or bolus, Zoetis) midazolam (0.1-0.3 mg kg^{-1} hr^{-1}, Avet Pharma), and/or ketamine (1-5 mg kg^{-1} hr^{-1} or bolus, Covetrus). Prior to ARDS induction, a bolus of 100 units kg^{-1} of heparin sodium (Pfizer) was given and a continuous infusion started at 100 units kg^{-1} hr^{-1}, titrated to activated clotting time (ACT) of 150-200 (Hemochron). Animals were maintained on 0.9% NaCl, Lactated Ringer's Solution, or 5% dextrose as needed based on fluid balance and blood glucose measurements.

ARDS induction

Gastric aspiration injury was induced by previously established injury methods.[1–4] Briefly, the tip of a bronchoscope (Ambu® aScope™ 4 Broncho Slim 3.8/1.2) was positioned sequentially 1cm distal to the carina into the right and left mainstem bronchi and standardized gastric contents (30-50 mL; pH 2) were delivered to the bilateral lungs. The location of the tip of the bronchoscope was confirmed visually prior to delivery of gastric contents. After delivery, gastric contents remained in the lungs and bronchial alveolar lavage was not performed to allow development of acute lung injury. Lipopolysaccharide (LPS) from *Escherichia coli* O55:B5 (10 µg kg^{-1}, Sigma) in 100 mL normal saline was infused via central intravenous catheter over 30 to 60 minutes. ARDS 0hr was defined as the timepoint at which the Pa_{O2}/FI_{O2} ratio was first less than 150 mmHg following LPS infusion.

Lung protective ventilation

Initial ventilator settings were tidal volume (TV) 10-12 mL kg^{-1}, positive end expiratory pressure (PEEP) of 5 cmH_2O, respiratory rate (RR) 12-20 breaths min^{-1}, and fraction of inspired oxygen (FI_{O2}) of 100%. After ARDS 0hr, ventilator settings were adjusted to ARDSNet parameters (TV 6 mL kg^{-1}, PEEP and Fi_{O2} per ARDSNet "Higher FI_{O2}" table, and RR titrated to maintain pH > 7.3 with permissive hypercapnia) (**Table S4**). PEEP and FI_{O2} were weaned as tolerated based on pulse oximetry and Pa_{O2}.

Use of paralytics and extracorporeal membrane oxygenation (ECMO)

Pancuronium bromide (initial bolus of 0.1 mg kg^{-1} followed by continuous infusion of 0.1-0.5 mg kg^{-1}, Hospira) was used for paralysis in animals with severe hypoxia or hypercarbia. Depth of anesthesia was verified by continuous hemodynamic monitoring and vital signs response to painful stimuli every 15 minutes from initiation of paralysis until 2 hours after cessation of the paralytic infusion.

ECMO was used as rescue therapy to attempt to prevent mortality in severely ill swine prior to planned study endpoint. Cannulation for Veno-Venous (VV)-ECMO was achieved via either 20Fr dual-lumen Avalon (Maquet) in the right external jugular vein, or with a 23Fr outflow cannula in the femoral vein and 17Fr inflow cannula in the

Disease Models & Mechanisms • Supplementary information

right external jugular vein. Veno-Arterial (VA)-ECMO was established with a 23Fr outflow cannula in the REJ and 15Fr arterial cannula in the femoral artery. In all cases, a centrifugal pump (Maquet Rotaflow) was used with an oxygenator and continuous data collection system (Viper).

Blood sample collection
Blood samples were drawn from a femoral arterial line for point-of-care analysis (Epocal). Additional samples were collected in test-specific specimen vials (BD Vacutainer) at predefined timepoints (**Figure 1**) to obtain complete blood count, basic metabolic panel, and liver function tests (Antech Diagnostics). Inflammatory markers (IFN-γ, IL-1α, IL-1β, IL-1rα, IL-2, IL-4, IL-6, IL-8, IL-10, IL-12, IL-18, and TNF-α) were analyzed in duplicate by the Discovery Assay Pig Cytokine Array (Eve Technologies). Other markers (D-Dimer, C-Reactive Protein, ferritin) were measured by commercially available enzyme-linked immunosorbent assays (ELISAs) per the manufacturer's instructions (**Table S5**).

Radiographic Assessment of Lung Edema (RALE) scoring
Chest radiographs were taken at baseline, following delivery of gastric contents, at ARDS 0hr, ARDS 6hr, ARDS 12hr, ARDS 18hr, ARDS 24hr, ARDS 36hr, ARDS 42hr, and ARDS46-48hr or if clinically indicated. Radiographs were randomly numbered, blinded, and delivered to two radiologists for review without reference to experimental timepoints. Each radiologist independently scored the radiographs based on the Radiographic Assessment of Lung Edema (RALE) which evaluates the extent of consolidation (0 → none, 1 → <25%, 2 → 25-50%, 3 → 50-75%, 4 → >75%) and density of alveolar opacities (1 → Hazy, 2 → Moderate, 3 → Dense) on a chest radiograph (**Table S6**).[5] To evaluate reliability of RALE scoring across two independent reviewers, Pearson's correlation coefficient was calculated. A Bland-Altman plot was used to visualize agreement between reviewers.

Experimental endpoint
Pre-defined experimental endpoints were ARDS 6hr (*n*=3) and up to ARDS 48hr. Animals were euthanized prior to experimental endpoint if their clinical status deteriorated despite maximal medical treatment, including vasopressor support, ECMO, and/or cardiopulmonary resuscitation per Advanced Cardiac Life Support, and in consultation with Columbia University Institute of Comparative Medicine veterinarians. Animals were euthanized with an overdose of pentobarbital sodium (100 mg kg^{-1}, Euthasol, Virbac). Death was confirmed via cessation of heartbeat, spontaneous respiration, and lack of corneal reflex by a veterinarian.

Histopathologic analysis of lung injury
Tissue samples were collected from lung segments were immediately fixed in cold phosphate-buffered 4% paraformaldehyde (ThermoScientific) for 24-48 h. Samples were then embedded in paraffin, sectioned at 3 μm or 5 μm thickness, and stained with hematoxylin and eosin (H&E) by the Department of Molecular Pathology at Columbia University Medical Center. H&E slides were randomly numbered prior to pathologic review by an experienced pulmonary pathologist under light microscopy without reference to experimental endpoints. Slides from normal swine lung tissue were included within the blinded set of slides for review. A previously described lung injury severity score which includes airway polymorphonuclear cells per high-power field (hpf), alveolar polymorphonuclear cells per hpf, alveolar edema, interstitial infiltrate (lymphocytes and neutrophils in the interstitium around vessels and airways and in alveolar septa and pleura), and interstitial edema (perivascular and peribronchial spaced expanded with edematous fluid) was applied (**Table S7**).[1]

Immunohistochemical staining
Lung sections were de-paraffinized, placed in boiling citrate buffer (pH 6.0) for antigen retrieval, and blocked with 10% normal goat serum in phosphate-buffered saline for 2 h at room temperature. Next, primary antibodies were diluted 1:100, applied, and incubated for 12 h at 4 °C or 4 h at room temperature. Secondary antibodies were diluted 1:200 and incubated for 1 h at room temperature. Sections were mounted in Vectashield Mounting Medium with DAPI (Sigma), and coverslips were applied. Images were obtained using an Olympus FSX100 microscope. Immunofluorescence stains for CD31 (Abcam), EpCAM (Abcam), pro-surfactant protein C (Abcam), P-selectin (Abcam), zonula occludens-1 (Abcam), and zonula occludens-3 (Abcam). A complete list of antibodies and dilutions used is provided in **Table S8**.

Disease Models & Mechanisms • Supplementary information

References

1. Guenthart BA, O'Neill JD, Kim J, et al. Regeneration of severely damaged lungs using an interventional cross-circulation platform. *Nature Communications 2019 10:1*. 2019;10(1):1-16. doi:10.1038/s41467-019-09908-1
2. Meers CM, Tsagkaropoulos S, Wauters S, et al. A Model of Ex Vivo Perfusion of Porcine Donor Lungs Injured by Gastric Aspiration: A Step Towards Pretransplant Reconditioning. *Journal of Surgical Research*. 2011;170(1):e159-e167. doi:10.1016/J.JSS.2011.05.015
3. Fraisse A, Bregeon F, Delpierre S, et al. Hemodynamics in experimental gastric juice induced aspiration pneumonitis. *Intensive Care Medicine 2006 33:2*. 2006;33(2):300-307. doi:10.1007/S00134-006-0457-2
4. Meers CM, de Wever W, Verbeken E, et al. A Porcine Model of Acute Lung Injury by Instillation of Gastric Fluid. *Journal of Surgical Research*. 2011;166(2):e195-e204. doi:10.1016/J.JSS.2010.10.015
5. Warren MA, Zhao Z, Koyama T, et al. Severity scoring of lung oedema on the chest radiograph is associated with clinical outcomes in ARDS. *Thorax*. 2018;73(9):840-846. doi:10.1136/thoraxjnl-2017-211280
